# Implementing Technology in Neuropsychological Assessments: A Scoping Review

**DOI:** 10.1007/s10916-026-02407-z

**Published:** 2026-05-08

**Authors:** Elton H. Lobo, Dashiell Young, Emily McCann, Deepa Sriram, Leander K. Mitchell, Deborah Brooks, Nadeeka N. Dissanayaka

**Affiliations:** 1 UQ Centre for Clinical Research, The University of Queensland, Royal Brisbane and Women’s Hospital campus, Herston, QLD 4029 Australia; 2 The School of Psychology, The University of Queensland, Brisbane, Australia

**Keywords:** Neuropsychological assessment, Technology, Implementation, Barriers, Facilitators

## Abstract

Neuropsychological assessments are traditionally conducted in person using standardised pen-and-paper methods. Technology has enabled computerised, tablet-based, and videoconference-based alternatives that improve accessibility for individuals in rural areas or with mobility issues. Despite promising developments, implementation has been slow. This scoping review was conducted to identify the barriers and facilitators associated with implementing technology in neuropsychological assessments. The review followed Arksey and O’Malley’s framework with Levac refinements and reported according to PRISMA-ScR guidelines. A comprehensive search was performed across eight electronic databases. Studies investigating technology-based neuropsychological or cognitive assessments examining barriers and/or facilitators in clinical and research settings were included. Two reviewers independently screened titles, abstracts, and full text. Data were analysed using the Theoretical Domains Framework (TDF). The review included 48 studies spanning 1998 to 2025. These studies were predominantly focused on Dementia and cognitive disorders. Findings were mapped across 13 of 14 TDF domains and synthesised into six themes: user capabilities, technological infrastructure, user experience design, support systems, interest holder perceptions and preferences, and implementation context. Barriers included technology anxiety, physical/cognitive impairments, poor connectivity, and clinician concerns about clinical observation. The identified facilitators include comprehensive training, technical reliability, personalised support systems, and consideration of interest holder perspectives. These findings highlight that successful implementation requires systematic approaches prioritising human and organisational factors beyond technology validation alone. Implementation efforts should emphasise capacity building and interest holder perspectives. Further research is needed to develop evidence-based frameworks focusing on co-design and systematic change management.

**Supplementary Information:**

The online version contains supplementary material available at 10.1007/s10916-026-02407-z.

## Introduction

Neuropsychological testing is a key diagnostic tool to identify the presence and severity of cognitive impairments [1]. Traditionally conducted by the gold standard pen-and-paper approach, neuropsychological assessments provide data across different cognitive domains (memory, executive function, attention, language, and visuoperception), neuropsychiatric and behavioural features [1]. This approach enables consistent administration and scoring, facilitating comparisons across patients, clinics, and timepoints.

Neuropsychological assessments are typically conducted in person. Access to such assessments, however, can be limited for individuals residing in rural and remote areas [2] and for those with mobility issues [3]. Advances in technology offer potential for neuropsychological assessment, with pen-and-paper tests now available in computer and mobile-based formats [4]. These digital assessments may facilitate more frequent testing, producing more reliable and informative longitudinal data while providing enhanced accessibility and cost-effectiveness [5]. Additionally, neuropsychological evaluations can be performed via videoconferencing, with recent research indicating that performance was equivalent to traditional face-to-face assessments [6]. Given that nearly half of neuropsychologists in the United States utilise videoconference-based neuropsychology [7] and that clients report high levels of acceptability towards this technology [8], technological innovations may enhance accessibility while preserving the accuracy of assessments.

While advancements in neuropsychology research through technology are promising, several limitations exist. Neuropsychologists have articulated concerns regarding modifications to standardised procedures and assessments, emphasising the need for further research to establish the psychometric equivalence between traditional and technology-based neuropsychological evaluations [9]. Additionally, the use of computer or video-conferencing for assessments has raised privacy concerns for both clinicians and patients, with data security regarded as a paramount concern [10]. Technology-enabled assessments are inherently dependent on advancing technologies [11], which vary in complexity from web-based evaluations to wearables and virtual reality [12]. This technological complexity introduces considerable variability concerning the types of technologies employed, the hardware and software available, and the cognitive demands associated with different digital assessments [13]. However, establishing psychometric equivalence represents only one dimension of the challenge. A parallel body of literature has identified implementation barriers. This includes clinician conservatism, workflow integration difficulties, and financial considerations as distinct obstacles to the uptake of technology-based assessment [14–16]. Such poor uptake is evidenced by findings that only 6% of instruments used by neuropsychologists are computerised despite decades of development [17]. This demonstrates that validation alone does not ensure adoption. Implementation considerations should encompass factors such as feasibility of integration into routine clinical practice, acceptability to practitioners and patients, cost-effectiveness, training requirements, and organisational readiness [15, 18, 19].

Given these recognised implementation challenges, there is a need to systematically synthesise the evidence on barriers and facilitators to guide effective technology adoption. Several systematic reviews have established psychometric equivalence between videoconference-based and traditional assessment methods [20, 21], and some have identified barriers and facilitators to technology adoption [22] and implementation [23]. However, these implementation factors have not been analysed using an established implementation science framework. The Theoretical Domains Framework (TDF) provides a comprehensive, theory-informed approach to identifying and categorising determinants of behaviour change [24], which can inform the development of targeted implementation strategies. Consequently, this scoping review aims to identify barriers and facilitators associated with the implementation of technology in neuropsychological assessments and to map these findings to the TDF to inform future implementation efforts.

## Methods

This scoping review was conducted to identify and synthesise evidence regarding barriers and facilitators for implementing technology to conduct neuropsychological assessments. The review followed the methodological framework outlined by Arksey and O’Malley [25] and incorporated refinements suggested by Levac and colleagues [26]. A scoping review approach was selected as it provides a systematic method for examining main concepts and knowledge gaps within emerging areas of research [25]. The review was reported according to the Preferred Reporting Items for Systematic Reviews and Meta-Analyses extension for Scoping Reviews (PRISMA-ScR) guidelines to ensure methodological transparency and reproducibility [27].

### Eligibility Criteria

Studies were considered eligible for inclusion if they investigated the implementation, adoption, or utilisation of technology-based neuropsychological or cognitive assessment tools. Further, it examined barriers, facilitators, or both related to their use in clinical or research settings. To be included, studies must have (i) consisted of or evaluated technology (including digitised versions of traditional tests, tablets/smartphones, videoconferencing platforms, mobile or web-based applications, virtual reality environments, gaming platforms, artificial intelligence-assisted systems, wearable sensing devices or telephone-based assessments) as a primary component of the assessment process; (ii) incorporated neuropsychological or cognitive assessments measuring domains such as memory, attention, executive function, processing speed, or language abilities; and (iii) presented primary empirical research findings. Only peer-reviewed articles published in English were considered for inclusion, with no geographical or temporal restrictions applied to capture the full breadth of available evidence.

Studies were excluded if they constituted systematic reviews, literature reviews, scoping reviews, narrative reviews, or meta-analyses, as the objective was to capture primary empirical evidence rather than synthesised findings from secondary research. Conference abstracts, dissertations, book chapters, editorials, and grey literature were excluded due to potential limitations in peer review processes, accessibility constraints, and insufficient methodological detail. Studies focusing solely on technology development, validation, or psychometric properties without consideration of implementation factors were excluded unless they also reported implementation-relevant data in their results section. Implementation-relevant data included empirical findings on barriers, facilitators, feasibility, acceptability, technical challenges, dropout or completion rates, clinician or patient feedback, or resource requirements. Studies that discussed implementation implications only in their discussion section without empirically collecting such data were excluded.

### Search Strategy

A comprehensive search strategy was developed in collaboration with an experienced health sciences librarian (LE) to identify relevant literature across multiple domains of technology implementation in neuropsychological assessment. The search strategy was designed to be highly sensitive to capture the breadth of evidence across this multidisciplinary field, encompassing literature from psychology, neuropsychology, health informatics, implementation science and technology domains.

The primary search was conducted across eight major electronic databases: MEDLINE, PsycINFO, Embase, CINAHL, Web of Science, Scopus, ACM Digital Library and IEEE Xplore Digital Library in January 2025. The complete search strategy for each database is provided in the supplementary material (Supplementary Appendix 1). Supplementary search strategies included manual screening of reference lists from included studies and relevant review articles to identify additional studies not captured through database searching.

### Selection of Evidence Sources

Following completion of the comprehensive database search, all identified records were imported into EndNote (Clarivate Analytics, US) referencing management software where automated duplicate detection and removal processes were applied, followed by transfer of the remaining citations to Covidence (Covidence, Australia) review software.

Prior to commencing full screening, a pilot screening was undertaken where two reviewers (DY and EL) independently screened a sample of fifty records, with subsequent discussion of any discrepancies to ensure consistent application of selection criteria. On completion, both reviewers independently conducted title and abstract screening of all remaining unique records against the inclusion and exclusion criteria, and knowledge gained through discussions.

Full-text articles were retrieved for all citations deemed potentially eligible during title and abstract screening. The same two independent reviewers then conducted detailed assessment of these full-text articles for final eligibility determination, with comprehensive documentation of reasons for exclusion using standardised categories. At both screening stages, disagreements between reviewers were resolved through structured discussion, with consultation of a third senior reviewer when consensus could not be achieved through initial discussion.

### Data Extraction

Data were extracted using a standardised form developed specifically for this review. Two reviewers (DY and EL) independently extracted author details, publication year, country, study design, participant characteristics, technology type, cognitive domains assessed, implementation context, barriers, facilitators and relevant outcomes from each included study. Disagreements between reviewers were resolved through discussion, with consultation of a third reviewer when consensus could not be reached.

### Data Analysis and Synthesis

After data extraction was completed, all information was entered into NVivo 14 (QSR International, USA) for further processing. Data analysis was conducted using content analysis method [28]. Information obtained from each included article were used to identify patterns and commonalities across studies. Barriers and facilitators were systematically organised into main themes and sub-themes through an iterative hybrid approach similar to the approach described by McGowan, Powell and French [29]. First, data were coded inductively to allow themes to emerge naturally from sentences and paragraphs within the extracted text. These emergent themes were then deductively mapped to the 14 domains of the Theoretical Domains Framework (TDF) [24]. One reviewer (EL) conducted coding independently holding regular discussion meetings with another reviewer (DS) to discuss emergent themes, verify coding decisions, and ensure consistent application of the TDF domains across all included studies. Additionally, descriptive statistics including frequencies and percentages were used to summarise study characteristics and findings in MS Excel (Microsoft, USA). The descriptive data obtained from the included studies were organised and presented in tables and figures according to the study objectives.

## Results

**Fig. 1:**
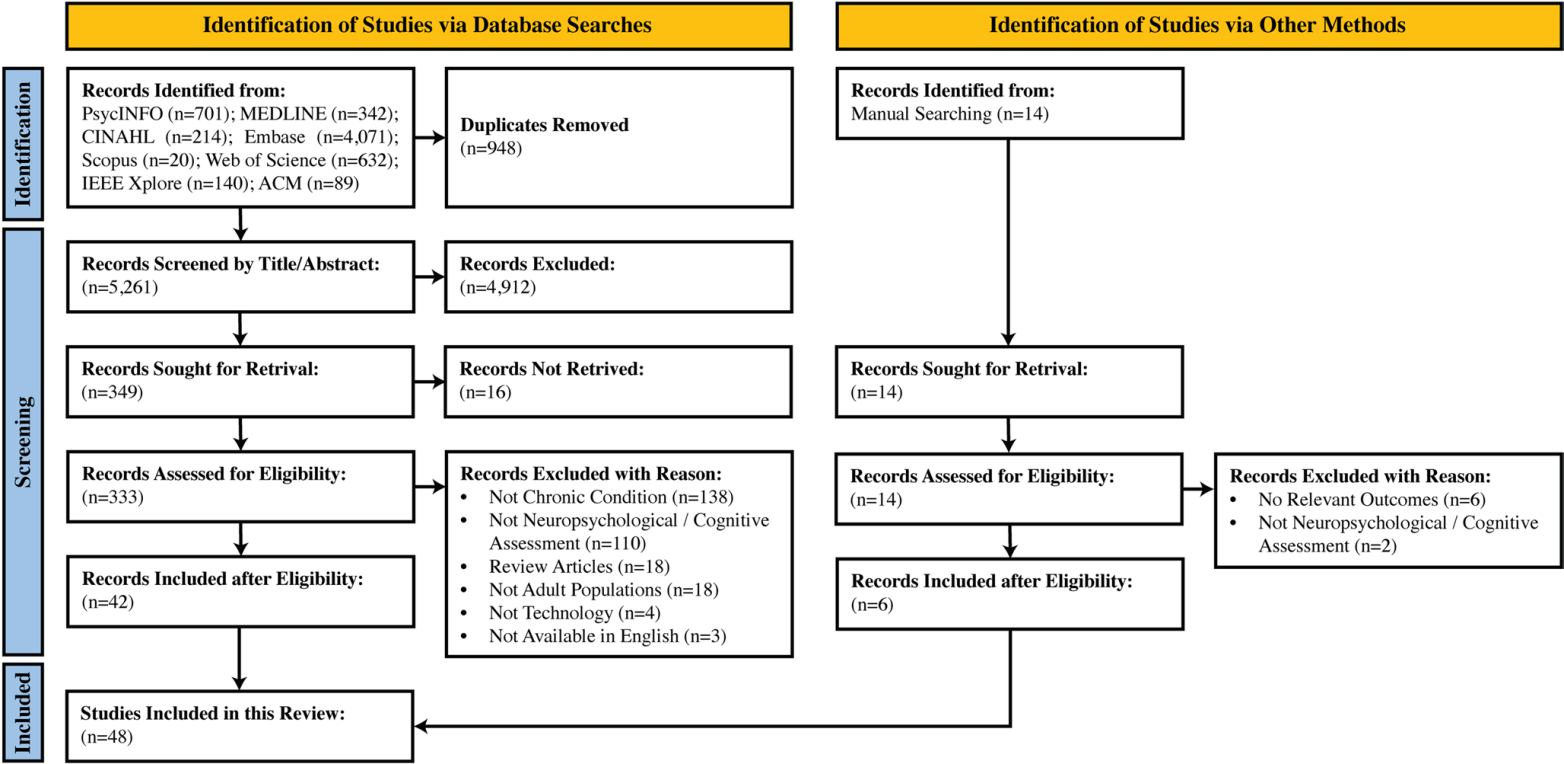
PRISMA Flow chart of the scoping review

A total of 6,209 records were obtained from the initial database search. After the removal of 948 duplicates, 5,261 records completed screening. Title and abstract screening excluded 4,912 records, identifying 349 full-text records for retrieval. Of these 349 records, 16 records could not be retrieved, resulting in 333 reports available for full-text review. Following full-text review against the inclusion and exclusion criteria, 291 records were deemed ineligible, yielding 42 included studies in this review. Manual screening of reference lists identified an additional six eligible articles, bringing the total number of included studies to 48 for this scoping review. Figure 1 illustrates the screening processes and reasons for exclusion, and Supplementary Appendix 2 consists of the list of included studies.

### Study Characteristics

**Fig. 2:**
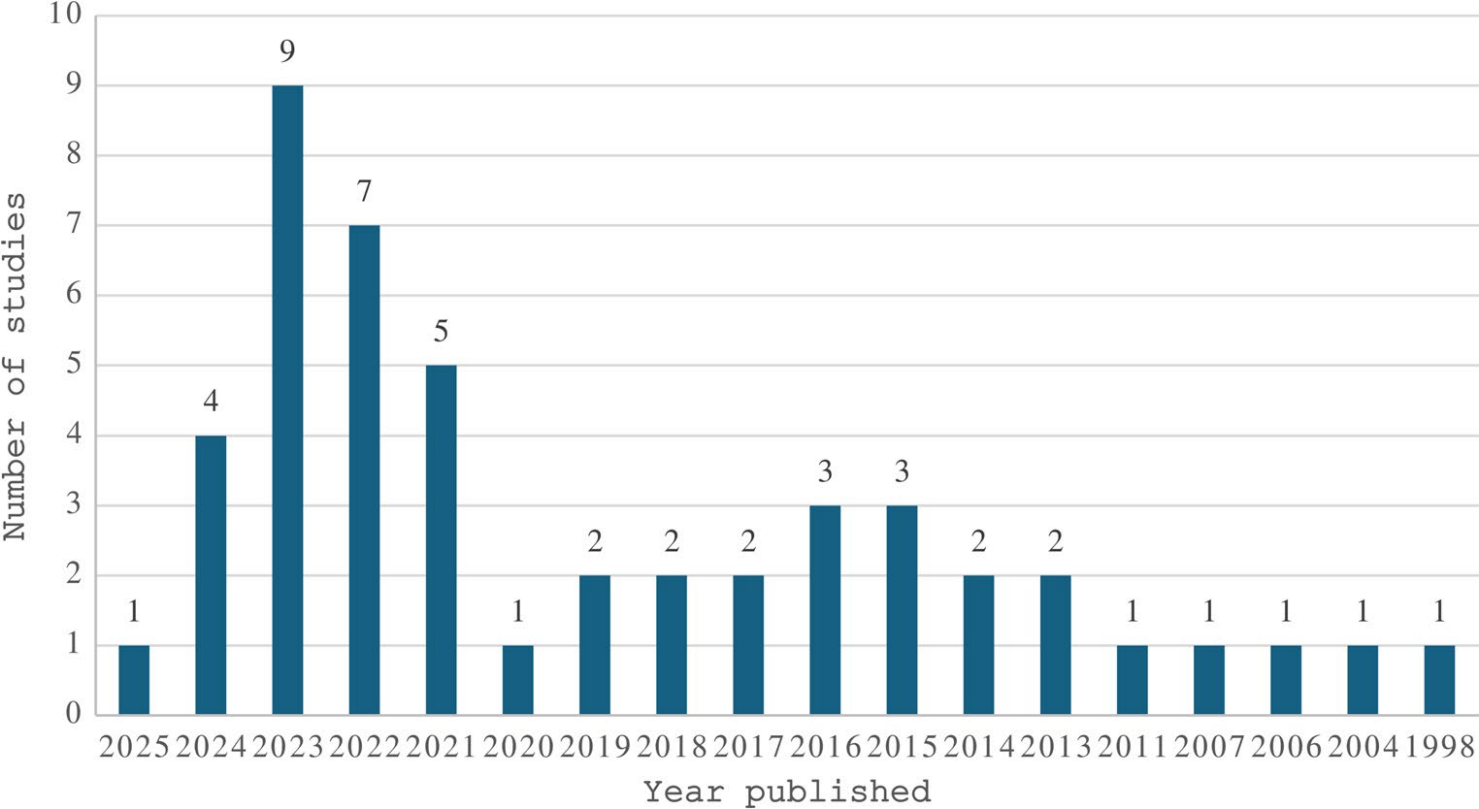
Number of studies published by year

**Fig. 3:**
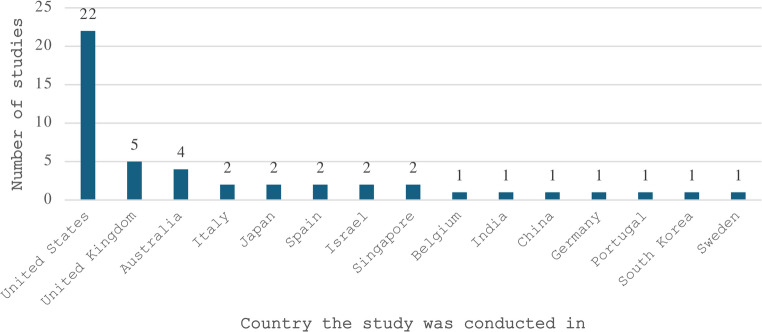
Number of studies by country

**Fig. 4:**
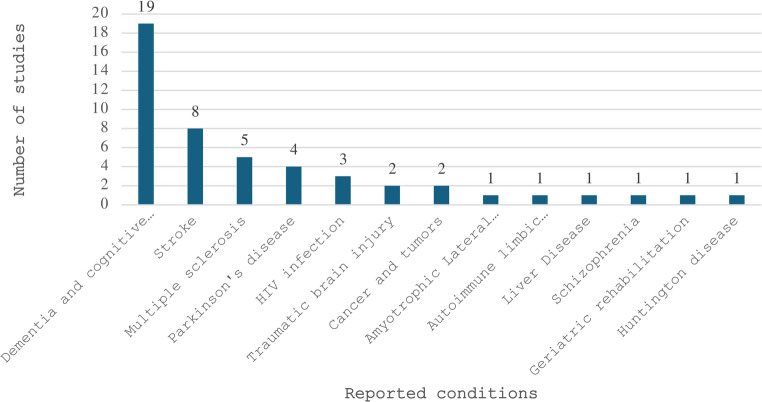
Number of studies by clinical conditions

The studies included in this scoping review spanned from 1998 to 2025 (Fig. 2), with the majority of research conducted between 2021 and 2023 (*n =* 21, 43.8%). Studies were conducted across 15 countries, with the United States representing nearly half of all studies (*n =* 22, 45.8%), followed by the United Kingdom (*n =* 5, 10.4%) and Australia (*n =* 4, 8.3%), with the remaining 12 countries presented in Fig. 3. Sample sizes ranged from 6 to 8,328, with reported ages ranging from 18 to 96 years, though one study did not report participant ages. Gender distribution showed a predominance of male participants (*n =* 27, 56.3%) compared to female participants (*n =* 17, 35.4%), with one study reporting equal gender distribution (2.1%) and three studies not reporting gender data (6.3%). Chronic clinical conditions were the most studied population, with dementia and cognitive disorders being the most prevalent (*n =* 19, 39.6%), followed by stroke (*n =* 8, 16.7%), multiple sclerosis (*n =* 5, 10%), and Parkinson’s disease (*n =* 4, 8.3%), with additional conditions represented by fewer studies as shown in Fig. 4. Recruited participants also included healthy or older adults in 17 studies (34%), clinicians in two studies (4%), and care partners in one study (2%).

### Technological Interventions

The characteristics of technology-based neuropsychological assessments across cognitive domains and technology platforms are presented in Table 1 and Supplementary Appendix 3. Global assessments were reported in 35 studies (72.9%) with *n* = 25 different instruments employed. The use of global assessments substantially exceeded any single cognitive domain; language was assessed by 13 studies (27.1%), followed by memory and executive function, each assessed by 11 studies (22.9%), while attention and processing speed were assessed in nine studies (18.8%). In terms of assessments, language had the highest number of individual assessments employed (*n* = 25), followed by memory and executive function (*n* = 14 each) and attention/processing speed (*n* = 9).

**Table 1:** Characteristics of technology-based neuropsychological assessments

Categories		Assessments, *n*	Studies, *n* (%)
Cognitive Domain(s)	Global	25	35 (72.9%)
	Language	25	13 (27.1%)
	Memory	14	11 (22.9%)
	Executive Function	14	11 (22.9%)
	Attention and Processing Speed	9	9 (18.8%)
	Emotional and Behavioural	8	6 (12.5%)
	Visual processing	6	6 (12.5%)
	Verbal Fluency	4	2 (4.2%)
	Mathematical/Calculation	1	1 (2.1%)
	Functional Abilities	1	1 (2.1%)
	Motor Abilities	1	1 (2.1%)
Physical Devices	Computer	57	20 (41.7%)
	Tablet	52	16 (33.3%)
	Smartphone	35	12 (25.0%)
	Telephone	24	12 (25.0%)
Application	Mobile Application	21	8 (16.7%)
	Web Application	4	5 (10.4%)
Delivery Approach	Videoconferencing	71	25 (52.1%)
	Gaming	7	2 (4.2%)
	VR	1	1 (2.1%)

Technology implementation demonstrated clear preferences across delivery methods and platforms. Videoconferencing dominated delivery approaches, comprising 71 individual assessments across 25 studies (52.1%). Gaming approaches were utilised in seven assessments across two studies (4.2%), while virtual reality appeared in only one assessment in one study (2.1%). Physical device utilisation showed computers being employed in 57 assessments and referenced in 20 studies (41.7%), followed by tablets in 52 assessments and 16 studies (33.3%), and smartphones (35 assessments) and telephones (24 assessments) each appearing in 12 studies (25.0%). Application types revealed mobile applications were the most common, encompassing 22 assessments and appearing in eight studies (16.7%), while web applications comprised 21 assessments but were only referenced in five studies (10.4%).

### Facilitators and Barriers

Table 2 presents the facilitators and barriers identified in the implementation of technology for neuropsychological assessments, mapped to the 14 domains of the Theoretical Domains Framework. Facilitators were identified across 12 of the 14 TDF domains, whereas barriers were identified in 9 domains. The domains of Social/Professional Role and Identity, Optimism, Intentions, and Goals contained no barriers. Additionally, Social Influences contained no facilitators, and Reinforcement contained neither barriers nor facilitators. Environmental Context and Resources demonstrated the highest frequency of both facilitators (*n* = 20) and barriers (*n* = 12). This domain was most frequently identified across studies, with facilitators referenced by 22 studies (45.8%) and barriers referenced by 24 studies (50%). It was followed by Beliefs about Consequences, with facilitators reported in 17 studies (35.4%) and barriers in 14 studies (29.2%), and Knowledge, with facilitators reported in 12 studies (25%) and barriers in 4 studies (8.3%).

**Table 2:** Barriers and facilitators in the implementation of technology for neuropsychological assessments mapped to theoretical domains framework

TDF Domain	Facilitators	Barriers
Knowledge	*For Individuals*: • Clear, comprehensible guidance facilitating successful technology use [30–33] • Understanding of ideal settings and conditions for technology-based assessments [34–37] *For Caregivers*: • Understanding of ideal settings and conditions for technology-based assessments [38] • Availability of training to establish and maintain optimal testing conditions [38] *For Clinicians and Healthcare Systems*: • Knowledge of technology capabilities [39, 40] and features for technology-based assessments [41]	*For Individuals*: • Inability to understand instructions [33, 34, 42, 43]
Skills	*For Individuals*: • Proficiency in digital skills facilitating and motivating engagement with technology-based assessments [44, 45] • Access to structured training and support for technology platform use [39, 46] *For Caregivers*: • Access to structured training and support for technology platform use [38] *For Clinicians*: • Proficiency in digital skills facilitating and motivating engagement with technology-based assessments [41] • Access to structured training and support for technology platform use [38]	*For Individuals*: • Insufficient digital literacy and limited familiarity with technology platforms causing difficulties in technology-based assessments [31, 45, 47] *For Caregivers*: • Insufficient digital literacy and limited familiarity with technology platforms causing delays in assessment [38]
Social/Professional Role and Identity	*For Clinicians*: • Maintain control and authority over assessment administration [41] and interpretation [39]	*No barriers identified*
Beliefs about Capabilities	*For Individuals*: • Possess the ability to operate technology-based assessments [46] without assistance [48]	*For Individuals*: • Insecurity and lack of confidence in one’s ability to use technology effectively [45]
Optimism	*For Individuals*: • Future cohorts will experience improved digital health interactions and literacy [41] *For Healthcare Systems*: • Increased integration of technology in future assessment practices [49–51]	*No barriers identified*
Beliefs about Consequences	*For Individuals*: • Technology based assessments are robust to individuals symptoms [52] • Optimal assessment requires balanced integration of automated and interpersonal elements [41] • Remote assessment removes transportation-related time [33, 47, 52–56], cost [49, 53, 54], and effort [47, 53, 57] *For Clinicians and Healthcare Systems*: • Technology based assessments are robust to individuals symptoms [58] and environmental factors [54] • Technology-mediated assessments are practical in real-world clinical settings [59] • Technology assessments decrease financial costs [59, 60] • Assessment results remain consistent regardless of technology modality used [61] • Technology-based assessments provide benefits in convenience, time, efficiency, clinical productivity, and overall workflow enhancement [30, 33, 37, 41, 53, 55, 62]	*For Individuals*: • Preference for in-person sessions [56, 63] and personal contact for assessments [64] • Technology failures validate superiority of face-to-face evaluation [56] • Cognitively intact individuals experience more distractions with remote assessment [45] *For Clinicians and Healthcare Systems* • Perceived superiority of in-person assessment [33] • Technology failures validate superiority of face-to-face evaluation [54] • Interruptions [65], pausing [66], and disturbances [36, 42, 47, 54, 55] in the testing environment disrupting assessment synchronicity, flow, and overall quality • Technology-induced performance misattribution [67] • Concerns related to self-administered assessments [46]
Reinforcement	*No facilitators identified*	*No barriers identified*
Intentions	*For Individuals*: • Preference to choose technology-mediated over traditional in-person assessment methods [68]	*No barriers identified*
Goals	*For Individuals*: • Technology assessments should be engaging [56] *For Healthcare Systems*: • Focus on assessment completion across multiple days rather than single sessions [68] • Technology assessments should be engaging [46, 69], and participants need to be entertained for long-term use [46]	*No barriers identified*
Memory, Attention and Decision Processes	*For Individuals*: • Ability to maintain focus and concentration throughout technology-based assessments [45]	*For Clinicians*: • Technology causing distraction during assessments [38]
Environmental Context and Resources	*For Individuals*: • Adequate internet bandwidth and connectivity required for effective technology-based assessment [52] • Technology-based tools should be economically sustainable and affordable [31, 70] • Intuitive interface design and user-friendly navigation [31–33, 41, 45, 46, 71] • Straightforward, uncomplicated technology without the need for specialised training or expertise [40, 52] • Availability of assistance and guidance for technology platform navigation [35, 41, 46] and connectivity issues [38] • Presence of caregivers or support persons to assist patients during technology-based assessments [38, 72] • Technology platforms must be adaptable to diverse clinical presentations and patient symptoms [41] • Technology features allowing customisation to individual needs and preferences [41] • Time allocation for technology-based assessments needs to be suitable and reasonable [34, 45] • Technology enabling broader access to assessments regardless of location or mobility [30, 31, 52] • Availability of equipment loan services to address technology access gaps [38] • Absence of environmental interruptions enabling assessment completion [45] • Adequate data protection and confidentiality safeguards [42] *For Clinicians and Healthcare Systems*: • Adequate internet bandwidth and connectivity required for effective technology-based assessment [52] • Absence of technology malfunctions or disruptions [64] • Technology functions consistently across different devices and systems [31, 37, 41] • Use of commonly available, non-specialised technology platforms [66, 70] • Technology-based tools should be economically sustainable and affordable [66] • Intuitive interface design and user-friendly navigation [33, 46] • Straightforward, uncomplicated technology without the need for specialised training or expertise [52] • Availability of assistance and guidance for technology platform navigation [37, 38] • Technology must maintain good validity across multiple device types [71, 73, 74] • Ease of incorporating technology-based assessments into existing clinical practices [55] • Efficient and immediate access to assessment reports and outcomes [41] • Need for controlled distraction-free settings for valid assessment administration [35, 38, 40]	*For Individuals*: • Broad range of technology-related problems and platform failures affecting assessment delivery [37, 38, 42, 55] • Lack of required devices and limited technology availability [45, 71] • Internet instability and connection issues [52, 55, 57] • Poor user interface design [31, 33, 47] and screen size limitations [70] • Clinical symptoms (e.g., tremor, cognitive impairment, visual and hearing impairment) interfering with technology use [41, 49, 53, 57, 58, 71, 75–77] • Limited time availability for completing technology-based assessments [63] • Clinical stage and disease severity affecting usability of the technology [34] • Frequency and duration of assessments perceived as excessive [45] *For Clinicians and Healthcare Systems*: • Broad range of technology-related problems and platform failures affecting assessment delivery [54, 57] • Internet instability and connection issues [31, 52, 57] • Loss of assessment data [31, 73] • Poor user interface design, screen size limitations [41], and compatibility issues [31, 52] • Technology platforms not suitable or adaptable for diverse populations [37] • Technology problems impacting assessment validity and outcomes [53, 64] • Technology cannot capture motor features adequately [75]
Social Influences	*No facilitators identified*	*For Individuals*: • Family members influencing participation in technology-based assessments [35]
Emotion	*For Individuals*: • Feelings of ease and confidence when interacting with digital assessment platforms [33, 37, 47, 55, 59, 68] • Favourable experience and satisfaction with technology-based assessment process [45, 47, 56, 63] • Technology-based assessments elicit feelings of interest, enjoyment, and engagement [46, 56, 69] *For Clinicians*: • Favourable experience with technology-based assessment process [33]	*For Individuals*: • Discomfort in technology-based assessments [33] • Frustration [38] and fatigue [37, 56, 63] when using digital assessment tools • Reduced motivation due to absence of in-person interaction [67] • Technology-induced anxiety negatively affecting assessment performance [56]
Behavioural Regulation	*For Individuals*: • Use of notifications and prompts as behavioural cues to maintain engagement and complete assessments [34] • Actively taking breaks to prevent exhaustion during assessments [37, 38]	*For Clinicians and Healthcare Professionals*: • Inability to ensure distraction-free testing environments [43]

**Fig. 5:**
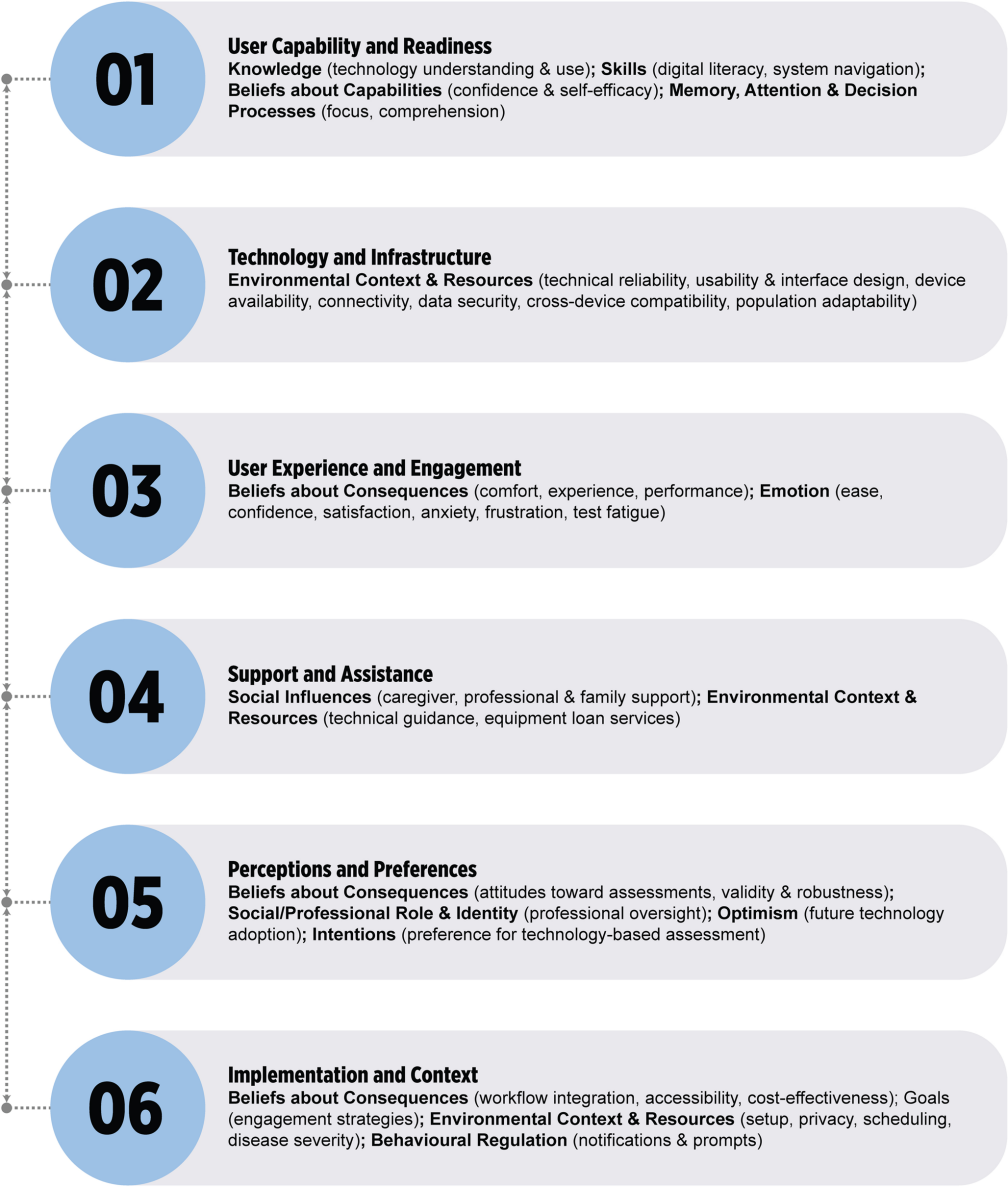
Themes identified based on the included studies

These facilitators and barriers were organised into six thematic categories as shown in Fig. 5: (i) *User Capability and Readiness*, which encompassed technological knowledge and skills, confidence and self-efficacy with technology, ability to maintain focus and concentration, and comprehension of instructions; (ii) *Technology and Infrastructure*, which included technical adequacy and reliability, system usability and interface design, equipment and device availability, internet connectivity, data security and confidentiality, compatibility across devices and systems, and adaptability to diverse populations; (iii) *User Experience and Engagement*, which comprised comfort and satisfaction with the assessment process, emotional responses such as ease, confidence, frustration, anxiety and fatigue, and engagement factors including interest and enjoyment; (iv) *Support and Assistance*, which covered technical support and guidance availability, caregiver and support person involvement, and family influences on participation; (v) *Perceptions and Preferences*, which addressed attitudes toward technology-mediated versus in-person assessment, beliefs about assessment validity and robustness across modalities, professional control, and intentions to choose technology-based methods; and (vi) *Implementation and Context*, which encompassed clinical workflow integration, accessibility regardless of location or mobility, cost-effectiveness and economic sustainability, environmental setup and distraction-free settings, customisation to individual needs and clinical presentations, assessment scheduling and time allocation, engagement strategies such as notifications and prompts, and the impact of clinical symptoms and disease severity on technology use.

## Discussion

The implementation of digital technologies in neuropsychological assessment represents a complex interplay of technical, human, and organisational factors that significantly influence adoption success. This is despite digital assessments’ perceived improvements over traditional assessments in accuracy, efficiency, and ease of use [78]. This necessitated this scoping review that included 48 studies spanning 1998–2025, classifying the barriers and facilitators into 13 out of 14 TDF domains. These barriers and facilitators were organised into six thematic categories, including (i) User Capability and Readiness; (ii) Technology and Infrastructure; (iii) User Experience and Engagement; (iv) Support and Assistance; (v) Perceptions and Preferences; and (vi) Implementation and Context.

### User Capability and Readiness

Digital literacy for all interest holders (i.e. individuals, caregivers and clinicians) emerge as a key determinant for successful implementation of neuropsychological assessments. Research literature consistently demonstrates that digital competence correlates with positive attitudes towards technology utilisation [79]. Significant gaps persist across both research findings and existing literature. Included studies in this review reveal limited technology experience among various interest holders [38, 41, 45], necessitating additional training [79–81] and requiring assistance and support for people with chronic conditions [31, 35]. The systematic review by Alotaibi, Wilson and Traynor [81] echoes these findings, with both empirical research [31, 33, 39, 41, 45–47, 52, 61, 72, 76] and literature [79–81] recommending user-friendly technology alongside adequate training and support to enhance digital competency.

However, given that age is the largest risk factor for multiple chronic conditions [82–84], it is important to note that several technological barriers associated with older adult populations warrant consideration. This includes technology anxiety from generational digital divides, cognitive decline, physical limitations, cybersecurity concerns, and insufficient tailored training and support [85]. Though, the characterisation of older age as an inherent barrier warrants nuance in light of recent evidence. Research during the COVID-19 pandemic highlighted the adaptive capacity of older adults when technology addresses essential needs. Telehealth use among older adults increased substantially during the pandemic [86], with video-based consultations rising from 4.6% to 21.1% among Medicare beneficiaries aged 70+ [87]. Importantly, when technology-enabling factors such as device ownership and internet access increased, age ceased to be a significant predictor of adoption [87]. This suggests that the digital divide, rather than age itself, constitutes the primary barrier [88]. Furthermore, generational cohort data suggest these barriers may be temporary rather than permanent features of ageing populations [89]. Successive waves of older adults demonstrate progressively higher baseline digital competence due to lifelong technology exposure [90], with older adults having accumulated workplace computer experience throughout their careers [91]. As digitally experienced cohorts replace earlier generations, technology barriers are projected to naturally diminish [89]. Accordingly, perceived usefulness and trust may serve as an influencing factor for technology adoption [92, 93]. For instance, Sun and Rau [94] demonstrated that people with chronic disease express willingness to use digital technologies when they perceive value in independently managing their disease. Another study highlighted the importance of trust as a fundamental concern among carers regarding technology acceptance, emphasising both the need for reliable intervention performance during critical moments and robust data security measures [93]. These findings highlight the critical need for technology design that considers the varying knowledge and skills among interest holders to ensure effective implementation in neuropsychological assessments.

### Technology and Infrastructure

Overcoming technical barriers and leveraging facilitators represent fundamental prerequisites for successful digital neuropsychological assessment. The findings demonstrated a diverse range of devices for neuropsychological assessment delivery, with most studies focusing on videoconference delivery systems. The emphasis on videoconferencing technology reflects its potential to improve access to care, patient satisfaction, convenience, device compatibility and cost-effectiveness [20, 95]. Further, it enables synchronous transfer of visual and auditory information required for neuropsychology service delivery [96]. However, adequate internet connectivity remains essential to maintain stable video connections [97], with enhanced security to protect the confidential sharing of personal information [98].

While bandwidth requirements are difficult to manage, especially in rural or regional areas [99], Brooks, Turvey and Augusterfer [98] propose the implementation of H.264 encoding standards to enhance high-definition video compression and minimise transmission interruptions, while maintaining compliance with the Health Insurance Portability and Accountability Act of 1996 (HIPAA) to protect sensitive data. Harris, Tang [100] supports this view, describing the need for proper encryption practices during collection, sharing and storage of data to ensure individual privacy and confidentiality. Beyond connectivity and security considerations, the infrastructure developed needs to ensure millisecond-level timing precision, as timing errors between intended and actual stimulus presentation generate noise rather than meaningful data [101]. These technical requirements align with those identified in the included studies towards the successful implementation of digital neuropsychological assessments.

### User Experience and Engagement

User experience and engagement represent critical determinants of long-term technology adoption, particularly as they promote positive emotions amongst diverse interest holders [45, 46, 56]. User acceptance has been found to correlate strongly with overall experience and graphical user interface clarity [102]. Partala and Saari [103] highlighted how negative emotional responses consistently led to unsuccessful technology adoptions. Research indicates several strategies for enhancing user experience, including extended participation periods with scheduled breaks to mitigate fatigue and test burden [34, 37, 45]. These considerations prove particularly vital for individuals with limited technological literacy [104] or those experiencing condition-related difficulties with keyboard navigation, password retention, and complex application usage [105].

The integration of gamified elements emerges as an alternative solution for maintaining data quality and enhancing user enjoyment, as evidenced by Lumsden, Skinner [106] and corroborated by two studies incorporating seven gaming-based assessments [34, 46]. In both strategies and/or solutions, developing such technology necessitates comprehensive validity assessments, precise target user identification, and individualised challenge optimisation to ensure therapeutic efficacy [107]. Co-design methodology represents an approach for developing neuropsychological assessments that genuinely address user needs and enhance engagement amongst people with Parkinson’s disease [108]. This partnership-based framework positions end users as primary intervention development drivers [109]. Such methodologies contribute to increased user satisfaction and promote acceptance, uptake, and long-term adherence [110–113]. This ultimately enables the successful implementation of a digital neuropsychological assessment that prioritises user experience and enjoyment.

### Support and Assistance

Support and assistance emerge as essential requirements for people with chronic conditions participating in digital neuropsychological assessments. This manifests through multiple modalities, including guidance from support staff [38, 41, 72] and caregivers [38, 72], alongside the provision of comprehensive instructions and protocols [53]. This necessity stems from multifaceted challenges encompassing individuals’ technological discomfort [33, 44], symptom difficulties [34, 39, 41, 71, 75, 76] and comprehension difficulties [38, 45] attributable to cognitive impairments [49]. For instance, motor symptoms in Parkinson’s Disease create significant challenges with input devices, as tremors impair precise mouse control and target selection, while touch screens require fine finger dexterity that many patients cannot achieve [114]. Similarly, people with cognitive impairment report difficulty in charging the device and caring for it [115]. Support provision thus proves essential for successful implementation, but careful consideration must be afforded to ensure caregivers do not inappropriately assist or prompt responses, thereby preserving assessment validity and diagnostic integrity [72].

### Perceptions and Preferences

Interest holder attitudes toward technology-mediated neuropsychological assessments represent a critical determinant of implementation success. Specifically, clinicians’ beliefs about assessment reliability and therapeutic relationships fundamentally shape adoption decisions [59, 64, 116]. This is due to the subtle nuances of clinical observation and rapport-building that have traditionally been considered essential for a valid assessment [117–119]. This perception is further supported by clinician reports of an inability to observe motor features and issues with technology misattributing individual performance [67, 75]. Clinicians’ concerns with maintaining control of the environment further complicate technology integration as this disrupts synchronicity, flow, and overall quality [36, 42, 47, 54, 55, 65, 66].

Despite the reported concerns, the field of neuropsychology is undergoing a transition, with younger clinicians demonstrating greater receptivity to technology integration [41]. The shift is driven by benefits including convenience, time savings, and improved clinical productivity and workflow [30, 33, 37, 41, 53, 55, 62] for all interest holders. Similarly, individuals prefer the reduced transportation time, costs, and burden associated with remote assessments [33, 47, 49, 52–57]. To manage these varying needs, the research literature consistently emphasises the importance of understanding the needs, values, and perceived usefulness of interest holders when developing digital health technologies [116, 120]. Such consideration is essential because perceptions often prove more influential than objective performance metrics [121, 122]. Understanding interest holder perspectives would complement the already promising research literature regarding the reliability of technology for delivering neuropsychological assessment [123]. This can be achieved through the aforementioned co-design methodology, further reinforcing its benefits. Therefore, striking a balance between interest holder needs and values, and the technology’s performance which is essential for successful implementation.

### Implementation and Context

Successful implementation of digital neuropsychological assessments requires careful consideration of contextual factors that influence both clinician adoption and operational effectiveness. Professional knowledge and confidence represent foundational elements, as clinicians who understand technological capabilities and advantages over traditional methods demonstrate greater implementation success [39–41]. The benefits of such assessments have been extensively discussed in the literature [78] and research findings [33, 47, 53, 54, 60, 62] as approaches to promote time saving, cost effectiveness, and increasing accuracy. Moreover, as healthcare becomes data-driven, such approaches can support and improve clinician decision-making and patient outcomes [78]. Concerns related to technical effects on assessment quality exist [41, 54, 56, 64, 70, 75] and can be an obstacle for implementation. These concerns are warranted, but research shows the reliability and agreement of various assessments delivered through technology when compared to in-person assessments [108]. Adaptations would be required for physical impairments [124] of the individual especially those with chronic conditions.

Other than knowledge and confidence in digital assessments, environmental considerations pose threats to the validity of the assessment process. Digital assessments need to be conducted in a distraction-free environment [36, 42, 43, 45, 47, 55], which can be achieved with in-person clinical settings [125]. Given that digital assessments are conducted in the home environment, ideal assessment environments are difficult to enforce by the clinician [43]. To establish a distraction-free environment, caregivers were included to support the clinicians [126]. Caregivers were instructed to eliminate auditory distractions such as turning off electronic devices, removing children and pets from the designated interview space, and silencing communication devices [126]. Such strategies need to be identified for the effective delivery of the assessment. At-home assessments have shown significant benefits for patients, including comfort [32, 54, 55, 64] at home environment [33, 47] and improved accessibility to neuropsychological services [127].

### Implications for Practice

The findings from this scoping review provide several evidence-based recommendations for clinical practice, classified across individual, organisational, and system implementation levels. At an individual level, training programmes for individuals, caregivers and clinicians should address both technical competencies and clinical adaptation skills. To maximise effectiveness, these programmes must include hands-on practice that closely simulates the actual working environment, with content tailored to participants ages and roles [128]. Research demonstrates that comprehensive training significantly increases user satisfaction with new technologies [128]. However, formal training alone may not be sufficient to address the ongoing practical challenges faced by individuals with chronic conditions during assessments. In these instances, the inclusion of caregivers in the assessment process can further support clinicians in delivery [126], and can support individuals in managing technical complexities [38, 72]. Test selection should focus on measures with established validity for remote administration [6]. Clinicians should nonetheless exercise caution in their selection, as reliability and accuracy may vary across populations, cognitive status, and assessment contexts [18, 129–132].

At an organisational level, technical implementation should prioritise HIPAA-compliant platforms for data access and privacy [133], reliable broadband [52], and dedicated technical support systems [38] to prevent failure of the infrastructure. To address digital literacy disparities, systems need to be designed through co-design methodologies to support user needs and capabilities [110–112], while improving user satisfaction, acceptance, and long-term adherence [110–112]. Furthermore, at a system level, research literature suggests the need to (i) prioritise dependability over advanced features; (ii) view technology systemically, not as isolated tools; (iii) recognise that technology operates within nested organisational routines; (iv) innovate incrementally, respecting existing systems; (v) critically examine standards and their underlying interests, and (vi) allow flexibility for local adaptation, when introducing new technologies to health services [134].

### Strengths and Limitations

This scoping review demonstrates several methodological strengths that enhance the breadth and depth of findings. The systematic approach followed established methodological frameworks and reporting guidelines to ensure transparency and reproducibility. The search strategy was comprehensive, encompassing eight major electronic databases across multiple disciplines, including psychology, neuropsychology, health informatics, and technology domains. The strategy was developed in collaboration with an experienced health sciences librarian. Rigorous screening procedures were implemented throughout the review process, including pilot testing between reviewers, dual independent screening at both title/abstract and full-text stages, and manual reference list screening that identified six additional eligible studies. The large evidence base of 48 studies spanning 1998–2025 provides substantial breadth across diverse clinical conditions, technologies, and implementation contexts. The systematic organisation of findings utilised the TDF, enabling structured identification of implementation factors across 13 of 14 domains. 4.

This review has several limitations that may affect the interpretation of findings. The search strategy was restricted to English-language peer-reviewed publications, potentially excluding valuable insights from grey literature and non-English studies. Given the applied nature of implementation research, the exclusion of grey literature represents a further notable limitation, as such sources often contain practical implementation experiences, organisational reports, quality improvement initiatives, and contextual factors that may not be captured in peer-reviewed publications. Real-world implementation insights, including operational challenges, adaptations made during deployment, and lessons learnt from failed implementations, are frequently documented in technical reports, white papers, and organisational documentation. This exclusion may have limited our understanding of the full spectrum of implementation barriers and facilitators encountered in routine clinical practice settings. Additionally, the exclusion of non-English articles may limit the diversity of cultural perspectives represented. No formal quality appraisal of included studies was undertaken, limiting conclusions about the strength and reliability of the evidence base. The categorisation of barriers and facilitators according to TDF domains involved subjective interpretation that may have produced different classifications with alternative reviewers. Some implementation factors may overlap across domains, and these overlaps may have been missed. Geographic bias toward United States studies (45.8%) may limit generalisability to other healthcare systems and cultural contexts, particularly regarding regulatory frameworks, reimbursement models, and healthcare delivery structures. The wide temporal span encompasses significant technological evolution, meaning some identified barriers may reflect outdated technologies rather than current implementation challenges. This may have resulted in overrepresentation of barriers that are less relevant in contemporary practice, while potentially underestimating emerging challenges associated with more sophisticated technologies. Consequently, findings should be interpreted recognising that technological capabilities, user expectations, and digital literacy have substantially evolved over this period. The heterogeneous mix of conditions and technologies may obscure specific factors requiring targeted attention in particular contexts.

## Conclusion

This scoping review found that successful implementation of technology in neuropsychological assessment requires coordinated attention to six interconnected themes, including user capabilities, technological infrastructure, user experience design, support systems, perception & preferences, and implementation context. The synthesis of 48 studies spanning nearly three decades demonstrates that technological capabilities have advanced dramatically, yet barriers still exist. Persistent barriers include technology anxiety among older adults, motor and cognitive impairments affecting device use, inadequate connectivity in rural areas, inability to control home testing environments, and clinician concerns about observing clinical features and maintaining therapeutic relationships. Addressing these barriers require comprehensive training, technical reliability, personalisation, and robust support systems, with inclusion of co-design methodologies to understand the interest holders’ perspectives and values in its design. These insights are particularly relevant as the field continues toward sustainable, evidence-based integration of digital neuropsychological assessment tools, emphasising the importance of human-centred design and systematic change management in healthcare technology adoption.

## Supplementary Information

Below is the link to the electronic supplementary material.

Supplementary Material 1 (PDF 41.1 KB)

Supplementary Material 2 (PDF 115 KB)

Supplementary Material 3 (PDF 72.1 KB)

## Data Availability

Not applicable.
