# Supplementary material for: Implementing Technology in Neuropsychological Assessments: A Scoping Review
Source: J Med Syst. 2026 May 8;50(1):73. doi: 10.1007/s10916-026-02407-z (PMC13156102; doi:10.1007/s10916-026-02407-z)
Supplement: Supplementary file 1 — Supplementary Material 1 (PDF 41.1 KB) [file 10916_2026_2407_MOESM1_ESM.pdf]

EBSCO (MEDLINE, PsycINFO, CINAHL)	
1	((MH Neuropsychology) OR (MH "Neuropsychological Tests") OR (MH "Stroop Test") OR (MH "Trail Making Test") OR (MH "Cognitive Dysfunction") OR (MH aphasia) OR (MH agnosia) OR (MH apraxias) OR (MH orientation) OR (MH "Orientation, Spatial") OR (MH "Depth Perception") OR (MH Attention) OR ((TI neuropsych* OR AB neuropsych*) OR (TI neurodegenerat* OR AB neurodegenerat*) OR (((TI cogniti* OR AB cogniti*) OR (TI neurocognit* OR AB neurocognit*)) N3 ((TI impair* OR AB impair*) OR (TI handicap* OR AB handicap*) OR (TI defect* OR AB defect*) OR (TI disorder* OR AB disorder*) OR (TI assess* OR AB assess*) OR (TI test* OR AB test*) OR (TI disabilit* OR AB disabilit*) OR (TI degenerat* OR AB degenerat*) OR (TI function* OR AB function*) OR (TI dysfunction* OR AB dysfunction*))) OR ((TI psycholog* OR AB psycholog*) N3 (TI tool* OR AB tool*)) OR (((TI memor* OR AB memor*) OR (TI attention* OR AB attention*) OR (TI executive-function* OR AB executive-function*) OR (TI language* OR AB language*) OR (TI perception* OR AB perception*) OR (TI visu*-construct* OR AB visu*-construct*)) N3 (TI test* OR AB test*)) OR (TI aphasi* OR AB aphasi*) OR (TI agnos* OR AB agnos*) OR (TI apraxi* OR AB apraxi*) OR (TI orientation OR AB orientation) OR (((TI depth OR AB depth) OR (TI visuospaci* OR AB visuospaci*)) N3 (TI perception OR AB perception)) OR ((TI mental* OR AB mental*) N3 ((TI concentrat* OR AB concentrat*) OR (TI speed OR AB speed))) OR ((TI processing OR AB processing) N3 (TI speed OR AB speed))))
2	("Diagnostic Techniques" AND (MH Procedures) OR (MH "Diagnostic Tests, Routine") OR ((((TI diagnos* OR AB diagnos*) OR (TI neuropsych* OR AB neuropsych*) OR (TI detect* OR AB detect*)) N3 ((TI assess* OR AB assess*) OR (TI test* OR AB test*) OR (TI procedure* OR AB procedure*) OR (TI accura* OR AB accura*) OR (TI validit* OR AB validit*) OR (TI reliab* OR AB reliab*)))) OR ((((TI memor* OR AB memor*) OR (TI attention* OR AB attention*) OR (TI executive-function* OR AB executive-function*) OR (TI language* OR AB language*) OR (TI perception* OR AB perception*) OR (TI visu*-construct* OR AB visu*-construct*)) N3 (TI test* OR AB test*))))
3	(MH "digital health+") OR "digital health" OR (MH telemedicine+) OR telemedicine OR (MH "e health+") OR "e health" OR "electronic health" OR (MH "m health+") OR "m health" OR "mobile health" OR "remote consultation" OR "digital transformation" OR "home care services" OR telenursing OR (MH "health innovation+") OR (MH telemetry+) OR (MH telehealth+) OR telehealth OR telecare OR (MH "digital care+")
4	1 AND 2 AND 3
**Embase**	
1	neuropsychology'/exp OR 'neuropsychological test'/exp OR 'stroop test'/exp OR 'trail making test'/exp OR 'cognitive defect'/exp OR 'aphasia'/exp OR 'agnosia'/exp OR 'apraxia'/exp OR 'orientation'/exp OR 'spatial orientation'/exp OR 'depth perception'/exp OR 'attention'/exp OR neuropsych*:ti,ab OR neurodegenerat*:ti,ab OR (((cogniti* OR neurocognit*) NEAR/3 (impair* OR handicap* OR defect* OR disorder* OR assess* OR test* OR disabilit* OR degenerat* OR function* OR dysfunction*)):ti,ab) OR ((psycholog* NEAR/3 tool*):ti,ab) OR (((memor* OR attention* OR 'executive function*' OR language* OR perception* OR 'visu* construct*') NEAR/3 test*):ti,ab) OR aphasi*:ti,ab OR agnos*:ti,ab OR apraxi*:ti,ab OR orientation:ti,ab OR (((depth OR visuospaci*) NEAR/3 perception):ti,ab) OR ((mental* NEAR/3 (concentrat* OR speed)):ti,ab) OR ((processing NEAR/3 speed):ti,ab)

2	diagnostic procedure'/exp OR 'diagnostic test'/exp OR ((diagnos* OR neuropsych* OR detect*) NEAR/3 (assess* OR test* OR procedure* OR accura* OR validit* OR reliab*)):ti,ab OR ((memor* OR attention* OR 'executive function*' OR language* OR perception* OR 'visu* construct*') NEAR/3 test*):ti,ab
3	'digital health'/exp OR 'digital health' OR telemedicine/exp OR telemedicine OR 'e health'/exp OR 'e health' OR 'electronic health' OR 'm health'/exp OR 'm health' OR 'mobile health' OR 'remote consultation' OR 'digital transformation' OR 'home care services' OR telenursing OR 'health innovation'/exp OR telemetry/exp OR telehealth/exp OR telehealth OR telecare OR 'digital care'/exp
4	1 AND 2 AND 3
**Scopus**	
1	(INDEXTERMS(Neuropsychology) OR INDEXTERMS("Neuropsychological Tests") OR INDEXTERMS("Stroop Test") OR INDEXTERMS("Trail Making Test") OR INDEXTERMS("Cognitive Dysfunction") OR INDEXTERMS(aphasia) OR INDEXTERMS(agnosia) OR INDEXTERMS(apraxias) OR INDEXTERMS(orientation) OR INDEXTERMS("Orientation, Spatial") OR INDEXTERMS("Depth Perception") OR INDEXTERMS(Attention) OR TITLE-ABS(neuropsych* OR neurodegenerat* OR ((cogniti* OR neurocognit*) W/3 (impair* OR handicap* OR defect* OR disorder* OR assess* OR test* OR disabilit* OR degenerat* OR function* OR dysfunction*)) OR (psycholog* W/3 tool*) OR ((memor* OR attention* OR executive-function* OR language* OR perception* OR visu*-construct*) W/3 test*) OR aphasi* OR agnos* OR apraxi* OR orientation OR ((depth OR visuospaci*) W/3 perception) OR (mental* W/3 (concentrat* OR speed)) OR (processing W/3 speed)))
2	("Diagnostic Techniques" AND INDEXTERMS(Procedures) OR INDEXTERMS("Diagnostic Tests, Routine") OR TITLE-ABS(((diagnos* OR neuropsych* OR detect*) W/3 (assess* OR test* OR procedure* OR accura* OR validit* OR reliab*))) OR TITLE-ABS(((memor* OR attention* OR executive-function* OR language* OR perception* OR visu*-construct*) W/3 test*)))
3	INDEXTERMS("digital health") OR "digital health" OR INDEXTERMS(telemedicine) OR telemedicine OR INDEXTERMS("e health") OR "e health" OR "electronic health" OR INDEXTERMS("m health") OR "m health" OR "mobile health" OR "remote consultation" OR "digital transformation" OR "home care services" OR telenursing OR INDEXTERMS("health innovation") OR INDEXTERMS(telemetry) OR INDEXTERMS(telehealth) OR telehealth OR telecare OR INDEXTERMS("digital care")
4	1 AND 2 AND 3
**Web of Science**	

1	(ALL=Neuropsychology OR ALL="Neuropsychological Tests" OR ALL="Stroop Test" OR ALL="Trail Making Test" OR ALL="Cognitive Dysfunction" OR ALL=aphasia OR ALL=agnosia OR ALL=apraxias OR ALL=orientation OR ALL="Orientation, Spatial" OR ALL="Depth Perception" OR ALL=Attention OR (TI=(neuropsych* OR neurodegenerat* OR ((cogniti* OR neurocognit*) NEAR/3 (impair* OR handicap* OR defect* OR disorder* OR assess* OR test* OR disabilit* OR degenerat* OR function* OR dysfunction*)) OR (psycholog* NEAR/3 tool*) OR ((memor* OR attention* OR executive-function* OR language* OR perception* OR visu*-construct*) NEAR/3 test*) OR aphasi* OR agnos* OR apraxi* OR orientation OR ((depth OR visuospaci*) NEAR/3 perception) OR (mental* NEAR/3 (concentrat* OR speed)) OR (processing NEAR/3 speed)) OR AB=(neuropsych* OR neurodegenerat* OR ((cogniti* OR neurocognit*) NEAR/3 (impair* OR handicap* OR defect* OR disorder* OR assess* OR test* OR disabilit* OR degenerat* OR function* OR dysfunction*)) OR (psycholog* NEAR/3 tool*) OR ((memor* OR attention* OR executive-function* OR language* OR perception* OR visu*-construct*) NEAR/3 test*) OR aphasi* OR agnos* OR apraxi* OR orientation OR ((depth OR visuospaci*) NEAR/3 perception) OR (mental* NEAR/3 (concentrat* OR speed)) OR (processing NEAR/3 speed))))
2	("Diagnostic Techniques" AND ALL=Procedures OR ALL="Diagnostic Tests, Routine" OR (TI=(((diagnos* OR neuropsych* OR detect*) NEAR/3 (assess* OR test* OR procedure* OR accura* OR validit* OR reliab*))) OR AB=(((diagnos* OR neuropsych* OR detect*) NEAR/3 (assess* OR test* OR procedure* OR accura* OR validit* OR reliab*)))) OR (TI=(((memor* OR attention* OR executive-function* OR language* OR perception* OR visu*-construct*) NEAR/3 test*)) OR AB=(((memor* OR attention* OR executive-function* OR language* OR perception* OR visu*-construct*) NEAR/3 test*))))
3	ALL="digital health" OR "digital health" OR ALL=telemedicine OR telemedicine OR ALL="e health" OR "e health" OR "electronic health" OR ALL="m health" OR "m health" OR "mobile health" OR "remote consultation" OR "digital transformation" OR "home care services" OR telenursing OR ALL="health innovation" OR ALL=telemetry OR ALL=telehealth OR telehealth OR telecare OR ALL="digital care"
4	1 AND 2 AND 3
**Engineering Journals (IEEE Xplore and ACM)**	
1	(Neuropsychology OR "Cognitive Dysfunction" OR "Stroop Test" OR "Trail Making Test" OR aphasia OR agnosia OR ((cognitive OR neurocognitive) AND (assessment OR function))) AND (("Diagnostic Tests" OR "Diagnostic Techniques") OR ((diagnos* OR neuropsych*) AND (assess* OR test* OR validit*)))
2	"digital health" OR telemedicine OR "e health" OR "electronic health" OR "m health" OR "mobile health" OR "remote consultation" OR "digital transformation" OR "home care services" OR telenursing OR "health innovation" OR telemetry OR telehealth OR telecare OR "digital care"
3	1 AND 2
