# Supplementary material for: Implementing Technology in Neuropsychological Assessments: A Scoping Review
Source: J Med Syst. 2026 May 8;50(1):73. doi: 10.1007/s10916-026-02407-z (PMC13156102; doi:10.1007/s10916-026-02407-z)
Supplement: Supplementary file 2 — Supplementary Material 2 (PDF 115 KB) [file 10916_2026_2407_MOESM2_ESM.pdf]

First Author	Year	Country	Title	Reported Conditions	Number of Participants	Gender of Participants	Age of Participants
Vijay Amarendran	2011	United States	The reliability of telepsychiatry for a neuropsychiatric assessment	Psychiatric disorders requiring neuropsychiatric assessment for tardive dyskinesia (schizophrenia, schizoaffective disorder, mood disorder)	Total patients: 50	Male: 47; Female: 3	Age range: 40 to 72 years
Debbie Gray	2024	United Kingdom	Development, reliability, validity, and acceptability of the remote administration of the Edinburgh Cognitive and Behavioural ALS Screen (ECAS)	Amyotrophic Lateral Sclerosis (ALS) / Motor Neurone Disease (MND)	Sub study a without ALS: 27; Sub study b with ALS: 24; Sub study c feedback clinicians: 19; Sub study c feedback patients: 17	Sub study a without ALS: 8 men, 19 women; Sub study b with ALS: 20 men, 4 women	Sub study a without ALS: Mean 35.2 years (SD 15.1, range 20-64 years); Sub study b with ALS: Mean 62.4 years (SD 12.6, range 31-85 years)
Isabel M. Monteiro	1998	United States	Reliability of routine clinical instruments for the assessment of Alzheimer's disease administered by telephone	Alzheimer's Disease and dementia	Total subjects: 30; Normal controls: 7; Mildly impaired: 8; Dementia patients: 15; Behave AD subgroup: 17	Total sample: 17 females, 13 males; Behave AD subgroup: 9 females, 8 males	Overall mean: 77.6±7.8 years (mean±SD); Females: 76.8±9.3 years (mean±SD); Males: 78.6±5.6 years (mean±SD); Behave AD subgroup mean: 78.3±6.5 years (mean±SD)
C. Munro Cullum	2006	United States	Feasibility of Telecognitive Assessment in Dementia	Dementia (Mild Cognitive Impairment and Alzheimer's disease)	Total: 33; Mild cognitive impairment: 14; Alzheimers disease: 19	Mild cognitive impairment: 9 men, 5 women; Alzheimers disease: 13 men, 6 women	Combined sample: 73.3±6.9 years (mean±SD), range 51-84 years
Michelangelo Dini	2025	Italy	Development and validation of an electronic Symbol-Digit Modalities Test for remote monitoring of people with multiple sclerosis	Multiple sclerosis	Total participants: 63	Total sample: 38 female, 25 male	Total sample: 52.5 years median (47-60.75 years interquartile range)
Mark Barber	2004	United Kingdom	Validity of the Telephone Interview for Cognitive Status (TICS) in post-stroke subjects	Stroke	Total: 64; Post stroke dementia: 24; No dementia: 40	Not reported	Median age: 72; Interquartile range: 63-80 years
Alexandra S. Atkins	2022	United States	Remote self-administration of digital cognitive tests using the Brief Assessment of Cognition: Feasibility, reliability, and sensitivity to subjective cognitive decline	Subjective cognitive decline (SCD)	Total older adults: 61; Healthy controls: 41; Subjective cognitive decline: 20	Healthy controls: 18 males (43.9%), 23 females (56.1%); Subjective cognitive decline: 7 males (35.0%), 13 females (65.0%)	Healthy controls: 67.02±7.71 years (mean±SD); Subjective cognitive decline: 70.30±9.76 years (mean±SD)
Paul Devos	2019	Belgium	Cognitive impairment screening using m-health: an android implementation of the mini-mental state examination (MMSE) using speech recognition	Cognitive impairment / Dementia screening	Total participants: 15	Females: 9; Males: 6	Average age: 86.5 years; Standard deviation: 5.95 years
N. J. Bourke	2023	United Kingdom	Assessing prospective and retrospective metacognitive accuracy following traumatic brain injury remotely across cognitive domains	Traumatic brain injury (TBI)	TBI patients: 111; Controls: 84	TBI patients: 82 men, 29 women; Controls: 41 men, 43 women	TBI patients: 45.32±14.15 years (mean±SD); Controls: 31.51±12.27 years (mean±SD)

Marina Sarno	2022	United States	Success of home-to-home tele-neuropsychology (TeleNP) in deep brain stimulation (DBS) candidacy assessments: COVID-19 and beyond	Parkinson's disease (patients being evaluated for Deep Brain Stimulation candidacy)	Total participants: 73	65.8% male overall	Mean age 63.3 years (SD = 8.75, range not specified)
Yoko Konagaya	2007	Japan	Validation of the Telephone Interview for Cognitive Status (TICS) in Japanese	Alzheimer's disease (AD)	Total: 135; Alzheimers disease patients: 49; Healthy controls: 86	Alzheimers patients: 19 men and 30 women; Healthy controls: 15 men and 71 women	Alzheimers patients: 75.2 ± 6.8 years (range 62-89); Healthy controls: 74.3 ± 7.2 years (range 60-90)
Garima Saini	2023	India	Video Teleconference Administration of the Addenbrooke's Cognitive Examination-III for the Assessment of Neuropsychological Status: An Experience in Indian Subjects with Cognitive Dysfunction	Cognitive dysfunction (dementia, mild cognitive impairment, subjective cognitive decline)	Total analyzed: 20	Males: 17; Females: 3; Percentage males: 85%	Total group: 62.7 ± 10.0 years (mean ± SD)
Aimee D. Brown	2024	Australia	Investigating Equivalence of In-Person and Telehealth-Based Neuropsychological Assessment Performance for Individuals Being Investigated for Younger Onset Dementia	Younger onset dementia (YOD)	Total participants: 43	Total participants: 21 females (48.8%), 22 males (51.2%)	Total participants: 60.26±7.19 years (mean±SD), range 38-71 years
Simona Raimo	2023	Italy	Comparing face-to-face and videoconference assessment of the Brief Repeatable Battery of Neuropsychological Tests in people with multiple sclerosis	Multiple sclerosis	Total participants: 60	Male: 38 (63.3%); Female: 22 (36.7%)	Mean age: 49.85±10.53 years; Disease duration: 14.54±9.83 years; Age at MS onset: 35.28±11.73 years
Jose M. Juarez	2014	Spain	Experiences on Computerised Neuropsychological Tests for Dementia Using a Mobile Touchable Interface	Dementia	Total: 6	Total: 3 males, 3 females	Mean: 71.1 years; Range: 55-83 years; Individual ages: 55, 76, 68, 83, 65, 80
Varna Jammula	2022	United States	The Montreal Cognitive Assessment (MoCA) in neuro-oncology: A pilot study of feasibility and utility in telehealth and in-person clinical assessments	Primary CNS tumors	Total: 71; In person: 47; Telehealth: 24	Total: 38 males (54%), 33 females (46%); In person: 24 males (51%), 23 females (49%); Telehealth: 14 males (58%), 10 females (42%)	Mean: 43 years (range 19-75); Overall range: 19-75 years
Gila Z. Reckess	2013	United States	Screening by telephone in the Alzheimer's disease anti-inflammatory prevention trial	Dementia and cognitive disorders (Alzheimer's disease prevention study)	Normal cognition: 189; Dementia: 11; MCI/pAD: 51; Other CIND: 25	Normal cognition: 53.40% male; Dementia: 72.70% male; MCI/pAD: 62.70% male; Other CIND: 48.00% male	Normal cognition: 78.46(3.97); Dementia: 79.64(4.32); MCI/pAD: 77.96(4.17); other CIND:77.40(3.12)
Tzvi Dwolatzky	2011	Israel	The mindstreams computerized assessment battery for cognitive impairment and dementia	Cognitive impairment and dementia (including Alzheimer's disease, mild cognitive impairment, and various neurological conditions)	Total participants: None	Gender: None	Age: None; Normative sample: Bimodal distribution with largest numbers between 20-30 and 70-80 years old
Jack Carson Taylor	2023	United States	Feasibility and acceptability of remote smartphone cognitive testing in frontotemporal dementia research	Frontotemporal dementia (FTD) spectrum disorders	Total combined studies: 214; Pilot study: 20; Main study: 194	Pilot study: 12 females (60%), 8 males (40%); Main study: 103 females (53.4%), 91 males (46.6%)	Pilot study: 64.5 ± 16 years (mean ± SD); Main study: 55.2 ± 15 years (mean ± SD), range 20-84 years

Sarah Levy	2023	United States	In-person and remote administrations of the symbol digit modalities test are interchangeable among persons with multiple sclerosis	Multiple Sclerosis (MS) and Clinically Isolated Syndrome (CIS)	Total: 212; In person follow up: 72; Remote follow up: 143	In person group: 48 women out of 72 (66.7%); Remote group: 105 women out of 143 (73.4%)	In person group: 44.2 ± 10.9 years; Remote group: 44.8 ± 11.8 years; Age range: 18 to 65 years
Kengo Shibata	2024	United Kingdom	Remote digital cognitive assessment reveals cognitive deficits related to hippocampal atrophy in autoimmune limbic encephalitis: a cross-sectional validation study	Autoimmune limbic encephalitis (ALE)	Healthy controls: 54; Patients with ALE: 21	ALE patients: 14 males, 7 females; Healthy controls: 23 males, 31 females	ALE patients: 63.19±8.10 years (mean±SD); Healthy controls: 65.56±7.31 years (mean±SD)
M Kohli	2023	United States	Concurrent validity and reliability of at-home teleneuropsychological evaluations among people with and without HIV	HIV infection	People with HIV: 80; People without HIV: 23	People with HIV: 66 men (83%), 14 women (17%); People without HIV: 18 men (78%), 5 women (22%); Total sample: 84 men (81.6%), 19 women (18.4%)	People with HIV: 58.7±11.0 years (mean±SD); People without HIV: 61.9±16.7 years (mean±SD)
Jairo A. Gonzalez	2022	United States	Preliminary findings from a telephone-based cognitive screening of an adult HIV research cohort during the COVID-19 pandemic	HIV infection with cognitive assessment during COVID-19 pandemic	Total cohort: 59	Female: 46% (27 out of 59); Male: 54% (32 out of 59)	Mean age: 61.3 years (SD = 6.9); Overall cohort: 61.0 years (SD = 7.7)
Xiangliang Chen	2015	China	Telephone-based cognitive screening for stroke patients in China	Stroke (acute ischemic stroke patients)	Total participants: 89	Total participants: 65.2% male (58 men, 31 women approximately); Cognitively impaired subgroup: 51.3% female vs 17.1% female in normal group	Mean age: 62.9 ± 8.6 years; Cognitively impaired: 64.2 ± 9.8 years; Cognitively normal: 61.0 ± 7.1 years
Tereza Stillerova	2016	Australia	Could everyday technology improve access to assessments? A pilot study on the feasibility of screening cognition in people with Parkinson's disease using the Montreal Cognitive Assessment via Internet videoconferencing	Parkinson's disease	Total participants: 11; Participants with parkinsons: 11	Men: 7; Women: 4	Median age: 69.0 years; Interquartile range: 57.0-76.0 years
Oscar Y. Franco-Rocha	2023	United States	Remote assessment of cognitive dysfunction in hematologic malignancies using web-based neuropsychological testing	Hematologic malignancies (Multiple Myeloma and Non-Hodgkin lymphoma)	Multiple myeloma (MM) or nonhodgkin lymphoma (NHL): 22; Healthy controls: 40	Cancer patients: 50% male; Controls: 52% male	Cancer patients: 59.19±11.87 years (mean±SD); Controls: 54.59±9.53 years (mean±SD)
Jennifer L. Thompson	2023	United States	Preliminary validity of a telephone-based neuropsychological battery in a consecutive series of persons with HIV disease referred for clinical evaluation	HIV Disease	Persons with HIV telephone: 59; HIV seronegative telephone: 44; Persons with HIV in person comparison: 41	PLWH telephone: 25.4% women; HIV seronegative telephone: 36.4% women; PLWH in person: 36.6% women	PLWH telephone: 49.7 (11.0) years, range 26–68; HIV seronegative telephone: 45.0 (18.0) years, range 19–77; PLWH in person: 49.8 (10.0) years, range 26–66
Erin Duricy	2022	United States	Comparing the reliability of virtual and in-person post-stroke neuropsychological assessment with language tasks	Post-stroke (left-hemisphere stroke patients with language impairments)	Total: 48; Fully in person condition: 24; Partially virtual condition: 24	Females: 21; Males: 27; Fully in person IH IL: 11 females, 13 males; Partially virtual VH IL: 10 females, 14 males	Total sample: 63.4 ± 12 years (mean ± SD); Fully in person IH IL: 65.81 ± 13.56 years; Partially virtual VH IL: 61.08 ± 9.70 years

Corrine Durisko	2016	United States	A flexible and integrated system for the remote acquisition of neuropsychological data in stroke research	Stroke	Total: 16; In laboratory: 8; At home: 8	Total: 10 men, 6 women; In laboratory: 5 men, 3 women; At home: 5 men, 3 women	Range: 50-79 years; In laboratory: 50-78 years; At home: 51-79 years
Montserrat Alegret	2021	Spain	From face-to-face to home-to-home: Validity of a teleneuropsychological battery	Alzheimer's disease, mild cognitive impairment, and mild dementia	Home to home NBACE: 338; Face to face NBACE: 7990	Face to face group: 63.1% female; Home to home group: 60.1% female	Face to face group: 74.3 years (SD 9.2), range 46-93; Home to home group: 74.0 years (SD 9.3), range 46-93
ME Lacruz	2013	Germany	Feasibility, internal consistency and covariates of TICS-m (telephone interview for cognitive status - Modified) in a population-based sample: Findings from the KORA-Age study	Cognitive impairment and dementia in older adults	Total completed tics m: 3578	Male: 49.2%; Female: 50.8%	Mean age: 73 years (SD 5.89); Age range: 65-94 years
Jill R Settle	2015	United States	Remote cognitive assessments for patients with multiple sclerosis: a feasibility study	Multiple sclerosis (MS)	Total initial: 24; Home testing subset: 20; Completed all phases: 24 patients completed live and remote-in-office, 20 completed home testing	Total: 8 males (33%), 16 females (67%); Live first group: 5 males, 7 females; Remote first group: 3 males, 9 females	Mean overall: Approximately 47 years; Live first group: 46.6 years (SD 10.3); Remote first group: 47.4 years (SD 9.5)
Jorge Oliveira	2014	Portugal	Cognitive assessment of stroke patients with mobile apps: A controlled study	Stroke	Stroke patients: 15; Healthy controls: 15; Total: 30	Stroke patients: 9 male out of 15 total (60% male); Healthy controls: Not explicitly stated, but described as age and education-matched	Stroke patients: Mean age 45.5 years (SD = 12.3); Healthy controls: Age-matched to stroke patients; Age range: 18-60 years old
Hae-yeon Park	2017	South Korea	Korean Version of the Mini-Mental State Examination Using Smartphone: A Validation Study	Stroke (ischemic or hemorrhagic stroke)	Total patients: 30; Ischemic stroke: 19; Hemorrhagic stroke: 11	Male: 20 patients (66.6%); Female: 10 patients (33.3%)	Mean age: 69.83 ± 12.95 years; Range: Not specified
Jared F. Benge	2021	United States	Rapid communication: Preliminary validation of a telephone adapted montreal cognitive assessment for the identification of mild cognitive impairment in Parkinson's disease	Parkinson's disease with mild cognitive impairment	Total PD patients: 21; PD with MCI: 9; PD without cognitive impairment: 12	MCI group: 5 men (55.6%), 4 women (44.4%); No cognitive diagnosis group: 6 men (50%), 6 women (50%)	MCI group: 73.22±5.80 years (mean±SD); No cognitive diagnosis group: 71.25±6.68 years (mean±SD)
Herb Howard C. Hernandez	2022	Singapore	Cognitive Assessment by Telemedicine: Reliability and Agreement between Face-to-Face and Remote Videoconference-Based Cognitive Tests in Older Adults Attending a Memory Clinic	Cognitive impairment and dementia	Total completed: 56; Dementia diagnosis: 27; No dementia: 29	Total participants: 31 females (55.4%), 25 males (44.6%)	Total participants: 76.0±5.4 years (mean±SD)
Jodie E. Chapman	2019	Australia	Comparing face-to-face and videoconference completion of the Montreal Cognitive Assessment (MoCA) in community-based survivors of stroke	Stroke survivors	Total completed: 48	Men: 26; Women: 22; Percentage men: 54.2%	Mean age: 64.6 years; Standard deviation: 10.1 years; Range: 35-88 years
Kristen Dams-O'Connor	2018	United States	The Feasibility of Telephone-Administered Cognitive Testing in Individuals 1 and 2 Years after Inpatient Rehabilitation for Traumatic Brain Injury	Moderate-to-severe traumatic brain injury (TBI)	Year 1 TBI patients: 463; Year 2 TBI patients: 386	Year 1: 336 men (72.7%), 126 women (27.3%); Year 2: 275 men (71.2%), 111 women (28.8%)	Year 1: 47.9±20.9 years (mean±SD); Year 2: 47.1±20.2 years (mean±SD)

Maï-Carmen Requena-Komuro	2022	United Kingdom	Remote versus face-to-face neuropsychological testing for dementia research: a comparative study in people with Alzheimer's disease, frontotemporal dementia and healthy older individuals	Multiple dementia types: Alzheimer's disease, behavioural variant frontotemporal dementia, semantic dementia, progressive non-fluent aphasia, logopenic aphasia	Remote patients total: 25; Face to face patients total: 64; Healthy controls: 10; Alzheimers disease remote: 8; Alzheimers disease face to face: 25; BvFTD remote: 3; BvFTD face to face: 12; Semantic dementia remote: 4; Semantic dementia face to face: 9; PNFA remote: 5; PNFA face to face: 12; LPA remote: 5; LPA face to face: 6	Healthy controls: 7 men, 3 women; Alzheimers disease remote: 5 men, 3 women; Alzheimers disease face to face: 15 men, 10 women; BvFTD remote: 3 men, 0 women; BvFTD face to face: 8 men, 4 women; Semantic dementia remote: 2 men, 2 women; Semantic dementia face to face: 7 men, 2 women; PNFA remote: 2 men, 3 women; PNFA face to face: 6 men, 6 women; LPA remote: 4 men, 1 woman; LPA face to face: 4 men, 2 women	Healthy controls remote: 74.0±4.1 years; Healthy controls face to face: 69.5±4.1 years (tested 3-4 years earlier); Alzheimers disease remote: 69.8±5.9 years; Alzheimers disease face to face: 70.3±2.9 years; BvFTD remote: 72.0±5.6 years; BvFTD face to face: 60.3±5.2 years; Semantic dementia remote: 58.3±9.3 years; Semantic dementia face to face: 67.1±5.2 years; PNFA remote: 68.2±6.4 years; PNFA face to face: 69.5±3.6 years; LPA remote: 71.6±4.7 years
Allison Lindauer	2017	United States	Dementia Care Comes Home: Patient and Caregiver Assessment via Telemedicine	Alzheimer's disease and related dementia (mild, moderate, and severe stages)	Total consented: 28; Patients with AD: 28; Caregivers: 28	Patients female: 61%; Caregivers female: 61%	Patients mean: 71.6 years (SD = 11.6, range 51-96); Caregivers mean: 65.3 years (SD = 9.6, range 38-79)
Amir Abdolahi	2016	United States	A feasibility study of conducting the Montreal Cognitive Assessment remotely in individuals with movement disorders	Movement disorders (Parkinson disease and Huntington disease)	Total movement disorders: 17; Parkinson disease: 8; Huntington disease: 9	Parkinson disease: 7 men, 1 woman (87.5% male); Huntington disease: 4 men, 5 women (55.6% female)	Parkinson disease: 65.1±11.9 years (mean±SD); Huntington disease: 57.7±12.6 years (mean±SD)
Jasmohan S Bajaj	2015	United States	Validation of EncephalApp, Smartphone-Based Stroop Test, for the Diagnosis of Covert Hepatic Encephalopathy	Liver cirrhosis with covert hepatic encephalopathy	Healthy controls: 114; Patients with cirrhosis: 167	Cirrhotic patients: 119 men, 48 women; Controls: 77 men, 37 women	Cirrhotic patients: 55±7 years (mean±SD); Controls: 54±6 years (mean±SD)
Leela A. Rao	2022	United States	The Reliability of Telepractice Administration of the Western Aphasia Battery-Revised in Persons With Primary Progressive Aphasia	Primary Progressive Aphasia (PPA)	Unique participants: 19	Not specified by participant subtype - only noted that all participants spoke english as primary language and identified as white/caucasian, non-hispanic/latinx	Age at assessment: 64.4±5.8 years (mean±SD), range 55-74; Age at symptom onset: 59.7±6.2 years (mean±SD), range 49-70
Zhiwei Zeng	2018	Singapore	Towards Long-term Tracking and Detection of Early Dementia: A Computerized Cognitive Test Battery with Gamification	Mild cognitive impairment (MCI) and early dementia detection	Doctors: 8; Healthy older adults: 5; Total: 13	Not specified	Older adults group: Above 60 years; Doctors group: Not specified
Bruno Biagianti	2019	United States	Development and testing of a web-based battery to remotely assess cognitive health in individuals with schizophrenia	Schizophrenia	Healthy controls: 283; Schizophrenia patients: 104	Healthy controls: 54.60% males; Schizophrenia patients: 84.40% males	Healthy controls: 27.7±8.04 years (mean±SD); Schizophrenia patients: 24.1±7.7 years (mean±SD)
Jodie E Chapman	2021	Australia	Comparing performance across in-person and videoconference-based administrations of common neuropsychological measures in community-based survivors of stroke	Stroke survivors	Total stroke survivors: 48	Stroke survivors: 26 men, 22 women (54.2% male)	Stroke survivors: 64.6±10.1 years (mean±SD), range 35-88 years

David Berron	2024	Sweden	Remote and unsupervised digital memory assessments can reliably detect cognitive impairment in Alzheimer's disease	Alzheimer's disease (cognitively unimpaired and mild cognitive impairment participants)	Total: 100; CU amyloid negative: 49; CU amyloid positive: 28; MCI amyloid negative: 9; MCI amyloid positive: 14	52% female overall	Overall mean: 64.5 (10.4) years; CU amyloid negative: 60.6 (10.9) years; CU amyloid positive: 69.8 (8.4) years; MCI amyloid negative: 64 (11.9) years; MCI amyloid positive: 67.8 (4.3) years
Kiyoko Iiboshi	2020	Japan	A validation study of the remotely administered Montreal Cognitive Assessment Tool in the elderly Japanese population	Mixed cognitive conditions: mild cognitive impairment (MCI), dementia (Alzheimer's disease and dementia with Lewy bodies), and healthy controls	Total analyzed: 73	Total sample: 36 men (49.3%), 37 women (50.7%); MCI: 5 men (33.3%), 10 women (66.7%); Dementia: 5 men (35.7%), 9 women (64.3%); Healthy controls: 26 men (59.1%), 18 women (40.9%)	Total sample: 76.3±7.5 years mean±SD; MCI: 81.4±6.8 years; Dementia: 80.8±6.1 years; Healthy controls: 73.2±6.6 years
Tehila Eilam-Stock	2021	United States	Remote administration of the symbol digit modalities test to individuals with multiple sclerosis is reliable: A short report	Multiple sclerosis (MS)	MS patients: 132	MS patients: Approximately 103 women (78%), 29 men (22%)	MS patients: 50.17±12.26 years (mean±SD), range 18-69 years
Limor Zadik	2023	Israel	Evaluation of Montreal Cognitive Assessment (MoCA) Administered via Videoconference	Geriatric rehabilitation patients (post-orthopedic procedures)	Total participants: 44	Females: 33; Males: 11; Percentage female: 75%; Percentage male: 25%	Median age: 83 years; Interquartile range: 76-87 years
