# Supplementary material for: Implementing Technology in Neuropsychological Assessments: A Scoping Review
Source: J Med Syst. 2026 May 8;50(1):73. doi: 10.1007/s10916-026-02407-z (PMC13156102; doi:10.1007/s10916-026-02407-z)
Supplement: Supplementary file 3 — Supplementary Material 3 (PDF 72.1 KB) [file 10916_2026_2407_MOESM3_ESM.pdf]

Cognitive Domain	Assessments	Physical Devices				Applications		Delivery			User	
		Computer	Tablet	Smartphone	Telephone	Mobile Application	Web Application	Videoconferencing	VR	Gaming	Individual	Caregiver
Memory	MDT-OS (Mnemonic Discrimination Task for Objects and Scenes)			x		x					x	
				David Berron		David Berron					David Berron	
	ORR (Objects-In-Room Recall)			x		x					x	
				David Berron		David Berron					David Berron	
	WAIS-IV Digit Span	x	x		x			x			x	
		Maï-Carmen Requena-Komuro	Maï-Carmen Requena-Komuro		Jennifer L. Thompson			Maï-Carmen Requena-Komuro; Marina Sarno			Maï-Carmen Requena-Komuro; Jennifer L. Thompson; Marina Sarno	
	CVLT-3 (California Verbal Learning Test-Third Edition)							x			x	
								Marina Sarno			Marina Sarno	
	WMS-IV Logical Memory	x						x			x	
		Marina Sarno; Aimee D. Brown						Aimee D. Brown			Marina Sarno; Aimee D. Brown	
	Rey Complex Figure Test (RCFT)	x						x			x	
		Jodie E. Chapman (8)						Jodie E. Chapman (8)			Jodie E. Chapman (8)	
	Wechsler Memory Scale – Fourth Edition (WMS-IV) Visual Reproduction	x						x			x	
		Jodie E. Chapman (8); Aimee D. Brown						Jodie E. Chapman (8); Aimee D. Brown			Jodie E. Chapman (8); Aimee D. Brown	
	Global Deterioration Scale (GDS)				x						x	
					Isabel M. Monteiro						Isabel M. Monteiro	
	Hopkins Verbal Learning Test–Revised (HVLT-R)	x	x	x	x			x			x	
		C. Munro Cullum; M Kohli; Jodie E. Chapman (8); Aimee D. Brown	M Kohli	M Kohli	Jairo A. Gonzalez; Jennifer L. Thompson			C. Munro Cullum; M Kohli; Jodie E. Chapman (8); Aimee D. Brown			C. Munro Cullum; M Kohli; Jodie E. Chapman (8); Aimee D. Brown; Jairo A. Gonzalez; Jennifer L. Thompson	
	Category Fluency Test (CF) - vegetables naming task				x						x	
					Yoko Konagaya						Yoko Konagaya	
	Paired Associates Learning (PAL)			x		x					x	
				N. J. Bourke		N. J. Bourke					N. J. Bourke	
	Rivermead Behavioral Memory Test (RBMT)				x						x	
					Gila Z. Reckess						Gila Z. Reckess	
	Wechsler Adult Intelligence Scale 3rd Edition (WAIS-III) Letter Number Sequencing	x	x	x				x			x	
		M Kohli	M Kohli	M Kohli				M Kohli			M Kohli	
	Paced Auditory Serial Addition Test (Channel 1)	x	x	x				x			x	
		M Kohli	M Kohli	M Kohli				M Kohli			M Kohli	
	RMT faces (Recognition Memory Test for faces)	x	x	x				x			x	
		Maï-Carmen Requena-Komuro	Maï-Carmen Requena-Komuro	Maï-Carmen Requena-Komuro				Maï-Carmen Requena-Komuro			Maï-Carmen Requena-Komuro	
Emotional and Behavioral	Geriatric Depression Scale (15-item version)	x	x					x			x	
		Allison Lindauer	Allison Lindauer					Allison Lindauer			Allison Lindauer	
	Geriatric Depression Scale (30-item version)				x						x	
					Gila Z. Reckess						Gila Z. Reckess	
	RMBPC (Revised Memory and Behavioral Problems Checklist) - behavioral symptoms assessment	x	x					x			x	
		Allison Lindauer	Allison Lindauer					Allison Lindauer			Allison Lindauer	
	Beck Depression Inventory-Second Edition (BDI-II)							x			x	
								Marina Sarno			Marina Sarno	
	Beck Anxiety Inventory (BAI)							x			x	
								Marina Sarno			Marina Sarno	
	Behavioral Pathology in Alzheimer's Disease Rating Scale (BEHAVE-AD)				x						x	
					Isabel M. Monteiro						Isabel M. Monteiro	
	Emotional discrimination (EMD)			x		x					x	
				N. J. Bourke		N. J. Bourke					N. J. Bourke	
	Emotional control task (EMC)			x		x					x	
				N. J. Bourke		N. J. Bourke					N. J. Bourke	
	Hospital Anxiety and Depression Scale (HADS)	x						x			x	
		Aimee D. Brown						Aimee D. Brown			Aimee D. Brown	
		x						x			x	

	WAIS-IV Digit Span	Jodie E. Chapman (8); Aimee D. Brown; C. Munro Cullum						Marina Sarno; Jodie E. Chapman (8); Aimee D. Brown; C. Munro Cullum			Marina Sarno; Jodie E. Chapman (8); Aimee D. Brown; C. Munro Cullum	
	Symbol Digit Modalities Test (SDMT)	x	x		x		x	x			x	
		Jodie E. Chapman (8); Michelangelo Dini; Sarah Levy; Aimee D. Brown	Sarah Levy		Tehila Eilam-Stock		Michelangelo Dini; Tehila Eilam-Stock	Jodie E. Chapman (8); Sarah Levy; Aimee D. Brown			Jodie E. Chapman (8); Michelangelo Dini; Sarah Levy; Aimee D. Brown; Tehila Eilam-StockRetry	
Attention and Processing Speed	Stroop test		x	x		x					x	
			Jasmohan S Bajaj	Jasmohan S Bajaj		Jasmohan S Bajaj					Jasmohan S Bajaj	
	Motor Control (MC)			x		x					x	
				N. J. Bourke		N. J. Bourke					N. J. Bourke	
	Simple reaction time (SRT)			x		x					x	
				N. J. Bourke		N. J. Bourke					N. J. Bourke	
	Choice Reaction Time (CRT)			x		x					x	
				N. J. Bourke		N. J. Bourke					N. J. Bourke	
	Target Detection (TD)			x		x					x	
				N. J. Bourke		N. J. Bourke					N. J. Bourke	
	Trail Making Test (TMT) - Part A and Part B		x			x					x	
			Jose M. Juarez			Jose M. Juarez					Jose M. Juarez	
	Wechsler Adult Intelligence Scale 3rd Edition (WAIS-III) Symbol Search	x	x	x				x			x	
		M Kohli	M Kohli	M Kohli				M Kohli			M Kohli	
Language	Boston Naming Test (BNT)	x	x	x				x			x	
		Jodie E. Chapman (8); C. Munro Cullum; Aimee D. Brown; M Kohli; Maï-Carmen Requena-Komuro	M Kohli; Maï-Carmen Requena-Komuro	M Kohli				Marina Sarno; Jodie E. Chapman (8); C. Munro Cullum; Aimee D. Brown; M Kohli; Maï-Carmen Requena-Komuro			Jodie E. Chapman (8); C. Munro Cullum; Aimee D. Brown; M Kohli; Maï-Carmen Requena-Komuro; Marina Sarno	
	COWAT (Controlled Oral Word-Association Test)				x			x			x	
					Marina Sarno			Jairo A. Gonzalez			Marina Sarno; Jairo A. Gonzalez	
	Philadelphia Naming Task (PNT)		x	x				x			x	
			Erin Duricy; Corrine Durisko	Erin Duricy				Erin Duricy; Corrine Durisko			Erin Duricy; Corrine Durisko	
	Pyramids and Palm Trees Test (PPT)		x	x				x			x	
			Erin Duricy; Corrine Durisko	Erin Duricy				Erin Duricy; Corrine Durisko			Erin Duricy; Corrine Durisko	
	Camel and Cactus Test (CC)		x	x				x			x	
			Erin Duricy; Corrine Durisko; Maï-Carmen Requena-Komuro	Erin Duricy				Erin Duricy; Corrine Durisko; Maï-Carmen Requena-Komuro			Erin Duricy; Corrine Durisko; Maï-Carmen Requena-Komuro	
	Word Naming Test (WN)		x	x				x			x	
			Erin Duricy	Erin Duricy				Erin Duricy			Erin Duricy	
	Nonword Naming Test (NWN)		x	x				x			x	
			Erin Duricy; Corrine Durisko	Erin Duricy				Erin Duricy; Corrine Durisko			Erin Duricy; Corrine Durisko	
	Peabody Picture Vocabulary Test (PPVT)		x	x				x			x	
			Erin Duricy; Corrine Durisko	Erin Duricy				Erin Duricy; Corrine Durisko			Erin Duricy; Corrine Durisko	
	Category Fluency (fruits and vegetables)	x						x			x	
		C. Munro Cullum						C. Munro Cullum			C. Munro Cullum	
	Category Fluency Animals	x	x		x			x			x	
		Maï-Carmen Requena-Komuro; Jodie E. Chapman (8)	Maï-Carmen Requena-Komuro		Jairo A. Gonzalez			Maï-Carmen Requena-Komuro; Jodie E. Chapman (8)			Maï-Carmen Requena-Komuro; Jodie E. Chapman (8); Jairo A. Gonzalez	
	Letter Fluency (FAS and CFL)	x						x			x	
		C. Munro Cullum						C. Munro Cullum			C. Munro Cullum	
	Pearson Clinical Assessment		x					x			x	
			Corrine Durisko					Corrine Durisko			Corrine Durisko	
	BPVS (British Picture Vocabulary Scale)	x	x					x			x	
		Maï-Carmen Requena-Komuro	Maï-Carmen Requena-Komuro					Maï-Carmen Requena-Komuro			Maï-Carmen Requena-Komuro	
	GNT (Graded Naming Test)	x	x					x			x	
		Maï-Carmen Requena-Komuro	Maï-Carmen Requena-Komuro					Maï-Carmen Requena-Komuro			Maï-Carmen Requena-Komuro	
		x	x					x			x	

	NART (National Adult Reading Test)	Maï-Carmen Requena-Komuro	Maï-Carmen Requena-Komuro					Maï-Carmen Requena-Komuro			Maï-Carmen Requena-Komuro	
	Concrete and abstract synonyms tests	x	x					x			x	
		Maï-Carmen Requena-Komuro	Maï-Carmen Requena-Komuro					Maï-Carmen Requena-Komuro			Maï-Carmen Requena-Komuro	
	Non-word reading	x	x					x			x	
		Maï-Carmen Requena-Komuro	Maï-Carmen Requena-Komuro					Maï-Carmen Requena-Komuro			Maï-Carmen Requena-Komuro	
	Regular reading	x	x					x			x	
		Maï-Carmen Requena-Komuro	Maï-Carmen Requena-Komuro					Maï-Carmen Requena-Komuro			Maï-Carmen Requena-Komuro	
	Irregular reading	x	x					x			x	
		Maï-Carmen Requena-Komuro	Maï-Carmen Requena-Komuro					Maï-Carmen Requena-Komuro			Maï-Carmen Requena-Komuro	
	Monosyllabic word repetition	x	x					x			x	
		Maï-Carmen Requena-Komuro	Maï-Carmen Requena-Komuro					Maï-Carmen Requena-Komuro			Maï-Carmen Requena-Komuro	
	Bisyllabic word repetition	x	x					x			x	
		Maï-Carmen Requena-Komuro	Maï-Carmen Requena-Komuro					Maï-Carmen Requena-Komuro			Maï-Carmen Requena-Komuro	
	Trisyllabic word repetition	x	x					x			x	
		Maï-Carmen Requena-Komuro	Maï-Carmen Requena-Komuro					Maï-Carmen Requena-Komuro			Maï-Carmen Requena-Komuro	
	Graded difficulty sentence repetition	x	x					x			x	
		Maï-Carmen Requena-Komuro	Maï-Carmen Requena-Komuro					Maï-Carmen Requena-Komuro			Maï-Carmen Requena-Komuro	
	Spoken sentences	x	x					x			x	
		Maï-Carmen Requena-Komuro	Maï-Carmen Requena-Komuro					Maï-Carmen Requena-Komuro			Maï-Carmen Requena-Komuro	
	PALPA tests (Psycholinguistic Assessments of Language Processing in Aphasia)	x	x					x			x	
		Maï-Carmen Requena-Komuro	Maï-Carmen Requena-Komuro					Maï-Carmen Requena-Komuro			Maï-Carmen Requena-Komuro	
Mathematical/ Calculation	GDA (Graded Difficulty Arithmetic test)	x	x					x			x	
		Maï-Carmen Requena-Komuro	Maï-Carmen Requena-Komuro					Maï-Carmen Requena-Komuro			Maï-Carmen Requena-Komuro	
Visual processing	WAIS-IV Matrix Reasoning							x			x	
								Marina Sarno			Marina Sarno	
	WAIS-IV Block Design	x						x			x	
		Jodie E. Chapman (8); Aimee D. Brown						Jodie E. Chapman (8); Aimee D. Brown			Jodie E. Chapman (8); Aimee D. Brown	
	Clock Drawing Test	x						x			x	
		Jodie E. Chapman (8); Aimee D. Brown						Jodie E. Chapman (8); Aimee D. Brown			Jodie E. Chapman (8); Aimee D. Brown	
	Simple Copy Test	x						x			x	
		C. Munro Cullum						C. Munro Cullum			C. Munro Cullum	
	Trail Making Test (TMT) - Part A and Part B		x			x					x	
			Jose M. Juarez			Jose M. Juarez					Jose M. Juarez	
	VOSP OD (Visual Object and Space Perception object decision task)	x	x					x			x	
		Maï-Carmen Requena-Komuro	Maï-Carmen Requena-Komuro					Maï-Carmen Requena-Komuro			Maï-Carmen Requena-Komuro	
	WAIS-IV Similarities	x						x			x	
		Jodie E. Chapman (8); Aimee D. Brown						Marina Sarno; Jodie E. Chapman (8); Aimee D. Brown			Marina Sarno; Jodie E. Chapman (8); Aimee D. Brown	
	Oral Trail Making Test (OTMT) Part B				x			x			x	
					Jennifer L. Thompson			Marina Sarno				
	Letter Fluency (FAS)	x	x					x			x	
		Jodie E. Chapman (8); Maï-Carmen Requena-Komuro	Maï-Carmen Requena-Komuro					Jodie E. Chapman (8); Maï-Carmen Requena-Komuro			Jodie E. Chapman (8); Maï-Carmen Requena-Komuro	
	Flanker test			x		x				x	x	
				Jack Carson Taylor		Jack Carson Taylor				Jack Carson Taylor	Jack Carson Taylor	
	2-back working memory test			x		x				x	x	
				Jack Carson Taylor		Jack Carson Taylor				Jack Carson Taylor	Jack Carson Taylor	
	Go/No-Go test			x		x				x	x	
				Jack Carson Taylor		Jack Carson Taylor				Jack Carson Taylor	Jack Carson Taylor	
	Wisconsin Card Sort Test			x		x				x	x	
Executive				Jack Carson Taylor		Jack Carson Taylor				Jack Carson Taylor	Jack Carson Taylor	

Function	Adaptive Memory Test			x		x				x	x	
				Jack Carson Taylor		Jack Carson Taylor				Jack Carson Taylor	Jack Carson Taylor	
	Clock Drawing Test	x						x			x	
		C. Munro Cullum						C. Munro Cullum			C. Munro Cullum	
	Stroop test	x	x	x		x		x		x	x	
		M Kohli; Jodie E. Chapman (8)	M Kohli; Jasmohan S Bajaj	M Kohli; Jasmohan S Bajaj; Jack Carson Taylor		Jasmohan S Bajaj; Jack Carson Taylor		M Kohli; Jodie E. Chapman (8)		Jack Carson Taylor	M Kohli; Jodie E. Chapman (8); Jasmohan S Bajaj; Jack Carson Taylor	
	Trail Making Task (Trails)	x	x	x		x		x			x	
		Aimee D. Brown; Jodie E. Chapman (8)	Jose M. Juarez	N. J. Bourke		N. J. Bourke; Jose M. Juarez		Aimee D. Brown; Jodie E. Chapman (8)			Aimee D. Brown; Jodie E. Chapman (8); Jose M. Juarez; N. J. Bourke	
	D-KEFS Colour Word Interference (Color Naming, Word Reading, Inhibition, Switching)	x						x			x	
		Aimee D. Brown						Aimee D. Brown			Aimee D. Brown	
	Action/verb fluency task				x						x	
					Jennifer L. Thompson						Jennifer L. Thompson	
	Category Switching from the Delis-Kaplan Executive Function System (D-KEFS)				x						x	
					Jennifer L. Thompson						Jennifer L. Thompson	
Functional Abilities	Functional Assessment Staging (FAST)				x						x	
					Isabel M. Monteiro						Isabel M. Monteiro	
Motor Abilities	Trail Making Test (TMT) - Part A and Part B		x			x					x	
			Jose M. Juarez			Jose M. Juarez					Jose M. Juarez	
Verbal Fluency	Generative verbal fluency (supermarket items in one minute)				x						x	
					Gila Z. Reckess						Gila Z. Reckess	
	Controlled Oral Word Fluency Test	x	x	x				x			x	
		M Kohli	M Kohli	M Kohli				M Kohli			M Kohli	
	Category Fluency (Animals)	x	x	x				x			x	
		M Kohli	M Kohli	M Kohli				M Kohli			M Kohli	
	Action (Verb) Fluency	x	x	x				x			x	
		M Kohli	M Kohli	M Kohli				M Kohli			M Kohli	
Global	MoCA (Montreal Cognitive Assessment)	x	x	x	x			x			x	
		Allison Lindauer; Tereza Stillerova; Amir Abdolahi; Jodie E Chapman (33); Kiyoko Iiboshi	Allison Lindauer; Tereza Stillerova; Varna Jammula	Tereza Stillerova; Limor Zadik	Jared F. Benge			Allison Lindauer; Marina Sarno; Tereza Stillerova; Limor Zadik; Varna Jammula; Amir Abdolahi; Jodie E Chapman (33); Kiyoko Iiboshi			Allison Lindauer; Tereza Stillerova; Amir Abdolahi; Jodie E Chapman (33); Kiyoko Iiboshi; Varna Jammula; Limor Zadik; Jared F. Benge; Marina Sarno	
	Mini-Mental State Examination (MMSE)	x	x	x	x	x		x			x	
		C. Munro Cullum; Aimee D. Brown	Hae-yeon Park	Paul Devos; Hae-yeon Park	Isabel M. Monteiro	Paul Devos		C. Munro Cullum; Aimee D. Brown; Hae-yeon Park; Herb Howard C. Hernandez			C. Munro Cullum; Aimee D. Brown; Hae-yeon Park; Paul Devos; Isabel M. Monteiro; Herb Howard C. Hernandez	
	Edinburgh Cognitive and Behavioural ALS Screen (ECAS)	x	x					x			x	x (behavioral symptoms component)
		Debbie Gray	Debbie Gray					Debbie Gray			Debbie Gray	Debbie Gray
	Test of Premorbid Function (TOPF)	x						x			x	
		Jodie E. Chapman (8); Aimee D. Brown						Jodie E. Chapman (8); Aimee D. Brown			Jodie E. Chapman (8); Aimee D. Brown	
	Addenbrooke's cognitive examination-III (ACE-III)							x			x	
								Garima Saini			Garima Saini	
	Brief Cognitive Rating Scale (BCRS)				x						x	
					Isabel M. Monteiro						Isabel M. Monteiro	
	TICSm (modified 13-item version of TICS)				x						x	
					Mark Barber; ME Lacruz						Mark Barber; ME Lacruz	
	TICS (Telephone Interview for Cognitive Status)				x						x	
					Mark Barber; Gila Z. Reckess; Yoko Konagaya						Mark Barber; Gila Z. Reckess; Yoko Konagaya	
	Self-administered Brief Assessment of Cognition		x			x					x	
			Alexandra S. Atkins			Alexandra S. Atkins					Alexandra S. Atkins	
	Mindstreams computerized cognitive tests	x					x				x	
		Tzvi Dwolatzky					Tzvi Dwolatzky				Tzvi Dwolatzky	
	Cognitron protocol battery	x					x				x	
		Kengo Shibata					Kengo Shibata				Kengo Shibata	
	[illegible]	x						x			x	

Global	BrainCheck - web-based cognitive testing battery	Oscar Y. Franco-Rocha						Oscar Y. Franco-Rocha			Oscar Y. Franco-Rocha	
	Systemic Lisbon Battery		x						x		x	
			Jorge Oliveira						Jorge Oliveira		Jorge Oliveira	
	Brief Test of Adult Cognition by Telephone (BTACT)				x						x	
					Kristen Dams-O'Connor						Kristen Dams-O'Connor	
	Virtual ADL+ House battery		x			x				x	x	
			Zhiwei Zeng			Zhiwei Zeng				Zhiwei Zeng	Zhiwei Zeng	
	Informant Questionnaire for Cognitive Decline in the Elderly (IQCODE) - 16-item version				x						x	
					Gila Z. Reckess						Gila Z. Reckess	
	5-minute NINDS-CSN protocol				x						x	
					Xiangliang Chen						Xiangliang Chen	
	Six-item screener (SIS)				x						x	
					Xiangliang Chen						Xiangliang Chen	
	AMT (Abbreviated Mental Test)							x			x	
								Herb Howard C. Hernandez			Herb Howard C. Hernandez	
	Neuropsychological battery of Fundacio ACE (NBACE)	x	x	x				x			x	
		Montserrat Alegret	Montserrat Alegret	Montserrat Alegret				Montserrat Alegret			Montserrat Alegret	
	CFAB (Chinese Frontal Assessment Battery)							x			x	
								Herb Howard C. Hernandez			Herb Howard C. Hernandez	
	WASI matrix reasoning (Wechsler Adult Intelligence Scale)	x	x					x			x	
		Maï-Carmen Requena-Komuro	Maï-Carmen Requena-Komuro					Maï-Carmen Requena-Komuro			Maï-Carmen Requena-Komuro	
	Rao's Brief Repeatable Battery of Neuropsychological Tests (R-BRB)							x			x	
								Simona Raimo			Simona Raimo	
	ANAM-MS (Automated Neuropsychological Assessment Metrics)	x						x			x	
		Jill R Settle						Jill R Settle			Jill R Settle	
	Online Neurocognitive Assessments (ONAs)	x					x				x	
		Bruno Biagianti					Bruno Biagianti				Bruno Biagianti	
	CDR (Clinical Dementia Rating Scale)	x	x					x				x
		Allison Lindauer	Allison Lindauer					Allison Lindauer				Allison Lindauer
